# Transcriptional Bursting in Gene Expression: Analytical Results for General Stochastic Models

**DOI:** 10.1371/journal.pcbi.1004292

**Published:** 2015-10-16

**Authors:** Niraj Kumar, Abhyudai Singh, Rahul V. Kulkarni

**Affiliations:** 1 Department of Physics, University of Massachusetts Boston, Boston, Massachusetts, United States of America; 2 Department of Electrical and Computer Engineering, University of Delaware, Newark, Delaware, United States of America; Rutgers University, UNITED STATES

## Abstract

Gene expression in individual cells is highly variable and sporadic, often resulting in the synthesis of mRNAs and proteins in bursts. Such bursting has important consequences for cell-fate decisions in diverse processes ranging from HIV-1 viral infections to stem-cell differentiation. It is generally assumed that bursts are geometrically distributed and that they arrive according to a Poisson process. On the other hand, recent single-cell experiments provide evidence for complex burst arrival processes, highlighting the need for analysis of more general stochastic models. To address this issue, we invoke a mapping between general stochastic models of gene expression and systems studied in queueing theory to derive exact analytical expressions for the moments associated with mRNA/protein steady-state distributions. These results are then used to derive noise signatures, i.e. explicit conditions based entirely on experimentally measurable quantities, that determine if the burst distributions deviate from the geometric distribution or if burst arrival deviates from a Poisson process. For non-Poisson arrivals, we develop approaches for accurate estimation of burst parameters. The proposed approaches can lead to new insights into transcriptional bursting based on measurements of steady-state mRNA/protein distributions.

## Author Summary

One of the fundamental problems in biology is understanding how phenotypic variations arise among individuals in a population. Recent research has shown that phenotypic variations can arise due to probabilistic cell-fate decisions driven by inherent randomness (noise) in the process of gene expression. One of the manifestations of such stochasticity in gene expression is the production of mRNAs and proteins in bursts. Bursting in gene expression is known to impact cell-fate in diverse systems ranging from latency in HIV-1 viral infections to cellular differentiation. Recent single-cell experiments provide evidence for complex arrival processes leading to bursting, however an analytical framework connecting such burst arrival processes with the corresponding higher moments of mRNA/protein distributions is currently lacking. We address this issue by invoking a mapping between general models of gene expression and systems studied in queueing theory. The framework developed and the results derived lead to new approaches for testing commonly used assumptions in modeling gene expression and for accurate estimation of burst parameters. Notably, the phenomenon of stochastic bursting has been observed in a wide range of disciplines ranging from neuroscience and finance to cell biology. The approaches developed and results obtained in this work will thus contribute towards quantitative characterization of burst processes in diverse systems of current interest.

## Introduction

The cellular response to fluctuating environments requires adjustments to cellular phenotypes driven by underlying changes in gene expression. Given the inherent stochasticity of cellular reactions, biological circuits controlling gene expression have to operate in the presence of significant noise [1–15]. While noise reduction and filtering is essential for several cellular processes [16], cells can also amplify and utilize intrinsic noise to generate phenotypic diversity that enables survival under stressful conditions [17]. Recent studies have demonstrated the importance of such bet-hedging survival strategies in diverse processes ranging from viral infections to bacterial competence [17]. Quantifying the kinetic mechanisms of gene expression that drive variations in a population of cells will thus contribute towards a fundamental understanding of cellular functions with important applications to human health.

Recent experiments focusing on gene expression at the single-cell level have revealed striking differences from the corresponding population-averaged behavior. In particular, it has been demonstrated that transcription in single cells is sporadic, with mRNA synthesis often occurring in bursts followed by variable periods of inactivity [7, 18–28]. Such transcriptional bursting can give rise to high variability in gene expression products and to phenotypic variations in a population of genetically identical cells [29–32]. Furthermore, dynamical parameters that characterize transcriptional bursting of key genes can significantly influence cell-fate decisions in diverse processes ranging from HIV-1 viral infections to stem-cell differentiation [17]. Correspondingly, there is significant interest in developing approaches for quantifying parameters related to transcriptional bursting such as frequency and mean burst size.

In recent years, multiple studies have provided evidence for bursty synthesis of mRNAs [20–25, 33, 34] and proteins [35, 36]. Experimental approaches in such studies include both steady-state measurements and time-dependent measurements of the mean and variance of gene expression products at the single-cell level. While obtaining time-lapse measurements of bursts at the single-cell level can be challenging, steady-state measurements at the single-cell level are now carried out routinely. It would thus be desirable to develop approaches for making inferences about burst parameters in gene expression using steady-state measurements at the single-cell level.

As noted in [37], steady-state measurements of the mean and variance alone cannot be used for estimating burst parameters for general models of gene expression, e.g. when burst arrival is governed by complex promoter-based regulation [38]. Additional insights into processes leading to transcriptional bursting can potentially be obtained using measurements of higher moments. However, analytical results for higher moments of steady-state mRNA and protein distributions in general models of expression have not been obtained so far. The derivation of the corresponding analytical expressions will elucidate how measurement of higher moments can potentially lead to quantification of burst parameters. To address these issues, it is essential to develop and analyze a general class of stochastic models of gene expression.

A simple stochastic model that is widely used in analyzing bursting in gene expression is the random telegraph model that takes into account the switching of promoter between transcriptionally active (ON) and inactive (OFF) states [39–41]. This model has been used as the basis for several studies focusing on inferring gene expression parameters based on observations of the mean and variance of mRNA/protein distributions [13, 27, 42]. In this model, in the limit that we have transcriptional bursting, the arrival of bursts is a Poisson process. Correspondingly, the waiting-time distribution between arrival of mRNA bursts is assumed to be exponential. In general, this assumption is not valid as there are multiple kinetic steps involved in promoter activation [37, 43, 44]. Recent experiments on mammalian genes [7, 45, 46] have demonstrated that the waiting-time for arrival of bursts does not have an exponential distribution. In view of these experimental observations, it is natural to ask: Using steady-state measurements, can we infer if the burst arrival process is *not* a Poisson process? If so, how can we estimate the corresponding burst parameters?

Furthermore, in estimating burst size it is commonly assumed that mRNA/protein bursts are geometrically distributed. This assumption, which has been validated by experimental observations for some genes, is derived from the corresponding distribution of bursts in the random telegraph model. However, given the complexity and diversity of gene expression mechanisms, it is possible that several promoters involve multiple rate-limiting steps in the transition from the ON state to the OFF state. In such cases, the transcriptional burst size distribution will not be a geometric distribution. This observation leads to the following question: Can we use steady-state measurements of moments to determine if the burst distribution deviates from a geometric distribution?

The aim of this paper is to address the above questions by considering models with general arrival processes for mRNA creation. The paper is organized as follows. First, we introduce a class of gene expression models with general arrival processes leading to mRNA/protein bursts with arbitrary burst distribution. Then we review the mapping from gene expression models to systems studied in queuing theory [43, 47, 48] and use this mapping to derive steady-state moments for mRNA/protein distributions. The analytical expressions obtained for the steady-state moments are used to develop approaches for estimating burst parameters for general arrival processes. Finally, we use the results obtained to derive conditions relating experimentally measurable quantities that determine if the arrival of mRNA bursts deviates from a Poisson process and if the distribution of mRNA bursts deviates from a geometric distribution.

## Results

### Model and preliminaries

**Fig 1:**
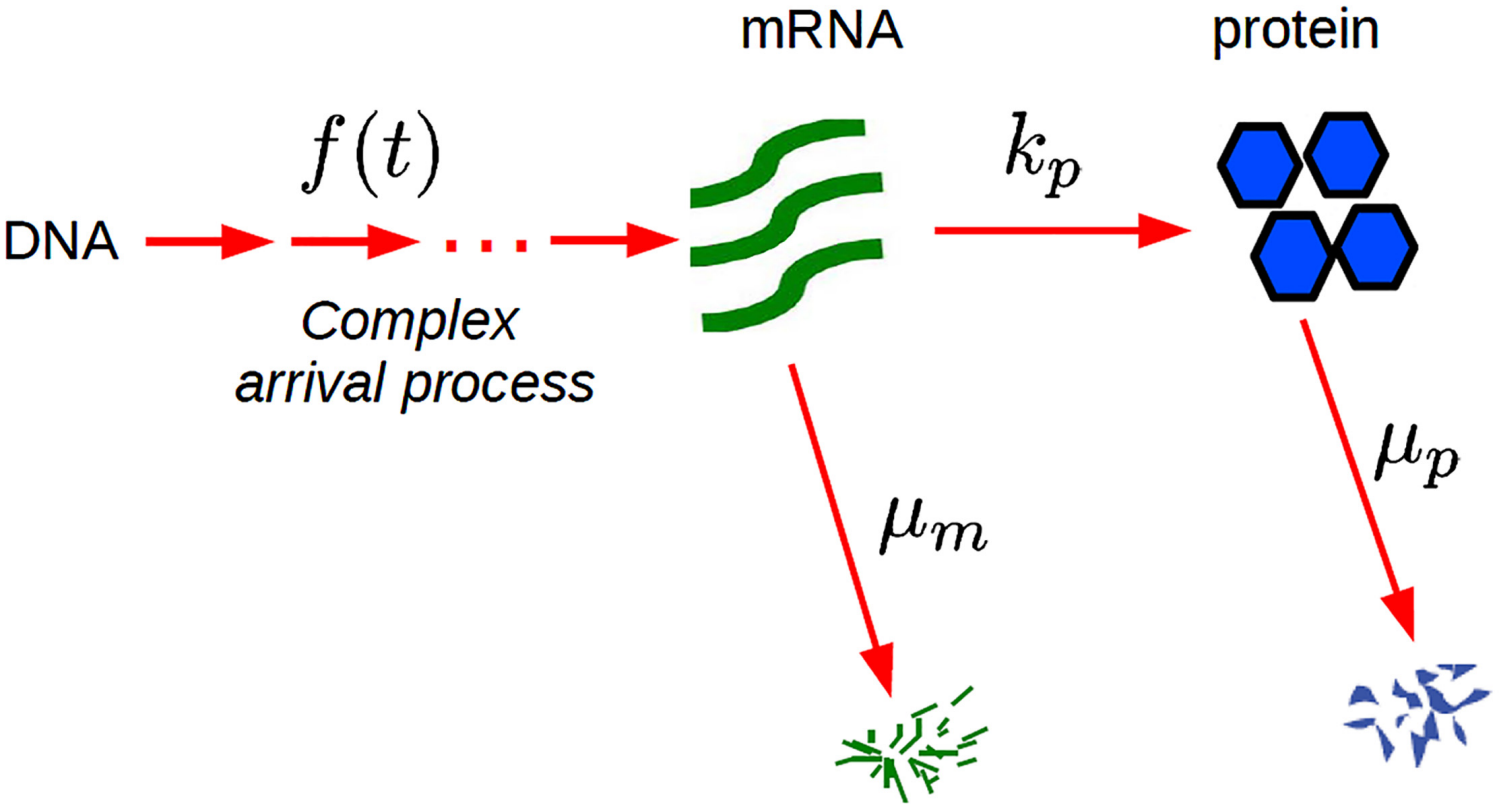
Kinetic scheme for the gene expression with general arrival time distributions. Bursts of mRNAs arrive with a general arrival time distributions *f*(*t*). Each mRNA produces proteins with rate *k*_*p*_ and mRNAs and proteins decay with rates *μ*_*m*_ and *μ*_*p*_, respectively.

We consider a general model of gene expression [43] as outlined in Fig 1. In the model, mRNAs are produced in bursts, with *f*(*t*) representing a general arrival time distribution for mRNA bursts. The mRNA burst distribution can be arbitrary. Each mRNA then produces proteins with rate *k*_*p*_, and finally, both mRNAs and proteins decay with rates *μ*_*m*_ and *μ*_*p*_, respectively. Note that the model also allows for post-transcriptional regulation since the protein burst distribution from each mRNA can be arbitrary; the only assumption is that each mRNA produces proteins independently.

In the limit *μ*_*p*_ ≪ *μ*_*m*_, we can use the bursty synthesis approximation [40] for analyzing protein dynamics. This approximation consists of two steps: 1) obtaining the distribution of proteins produced from each mRNA and 2) assuming that the proteins are produced in instantaneous bursts. The corresponding distribution for the number of proteins created is referred to as the protein burst distribution. A detailed justification of the validity of this approximation has been provided in previous work [40, 49].

Let $a^{p}\left(z\right)=\sum_{n=0}^{\infty }z^{n}p\left(n\right)$ denote the generating function of the protein burst distribution *p*(*n*) produced by a *single* mRNA, and let $A^{p}\left(z\right)=\sum_{n=0}^{\infty }z^{n}P\left(n\right)$ denote the generating function of the protein burst distribution *P*(*n*) produced by *all* the mRNAs in a burst. If we denote by *A* ^*m*^(*z*) the generating function of the mRNA burst distribution, then we have the following relation between the generating functions

$$A^{p}\left(z\right)=A^{m}[a^{p}\left(z\right)].$$ (1)

The above relation follows from the observation that the number of proteins produced in a burst is a compound random variable: the sum of *m* independent identical random variables, each of which corresponds to the number of proteins produced from a single mRNA in the burst and *m* itself is a random variable denoting the number of mRNAs produced in the burst.

While the analytical results that we derive are valid for general mRNA and protein burst distributions, we will primarily focus on a specific class of burst distributions. Simple kinetic models and the results from multiple experiments indicate that mRNA burst distributions are geometric [35]. Similarly, the burst distribution of proteins produced from a single mRNA is a geometric distribution with mean ⟨*p*_*b*_⟩ = *k*_*p*_/*μ*_*m*_. For a geometric distribution with mean ⟨*p*_*b*_⟩, the generating function is given by

$$a^{p}(z)=\frac{1}{\left[1+\langle p_{b}\rangle (1-z)\right]}.$$

If we condition the geometric distribution on the production of at least 1 mRNA, then the generating function for the corresponding conditional geometric distribution is given by

$$A^{m}(z)=\frac{z}{\left[1+\langle m_{b}\rangle (1-z)\right]}$$

with (1 + ⟨*m*_*b*_⟩) as the mean mRNA burst size. Note that in the limit ⟨*m*_*b*_⟩ → 0, this distribution reduces to exactly 1 mRNA produced in each burst. Thus the conditional geometric distribution provides a unified representation of both Poisson arrival process for mRNAs (⟨*m*_*b*_⟩ → 0) and processes leading to transcriptional bursting (⟨*m*_*b*_⟩ > 0).

Consider now the protein burst distribution produced by an underlying conditional geometric mRNA burst distribution with mean (1 + ⟨*m*_*b*_⟩). Using Eq (1), we see that the corresponding generating function of the protein burst distribution is given by

$$\begin{array}{c} A^{p}\left(z\right)=\frac{1}{1+\left\langle m_{b}\right\rangle \left\langle p_{b}\right\rangle \left(1-z\right)}. \end{array}$$

This is the generating function for a geometric distribution with mean *b* = (1 + ⟨*m*_*b*_⟩)⟨*p*_*b*_⟩), where ⟨*p*_*b*_⟩ = *k*_*p*_/*μ*_*p*_ represents the mean protein burst size from a single mRNA.

Single-cell experiments have demonstrated that the protein burst mean *b* can be directly measured in some cases [35]. However, if the protein production rate *k*_*p*_ is not known, the preceding analysis implies that measurements of protein burst distributions (which determine *b*) cannot be used to determine the degree of transcriptional bursting (1 + ⟨*m*_*b*_⟩). Since the mean transcriptional burst size is an important parameter characterizing bursting, it is of interest to develop approaches for estimating it based on available experiments. Previous work [50] has argued that the mean transcriptional burst size cannot be determined using measurements of protein burst distributions alone or by using only protein steady-state distributions. It was suggested that combining such measurements can potentially provide a way of determining the mean transcriptional burst size. To explore this possibility, it is necessary to derive analytical results connecting moments of burst and steady-state distributions for general kinetic schemes.

### Mapping to queueing theory: Results for moments

To obtain steady-state moments for the model outlined in Fig 1, we invoke the mapping of this gene expression model to systems studied in queueing theory [43, 48, 51, 52]. Broadly speaking, queueing theory is the mathematical theory of waiting lines formed by customers who, arriving according to some random protocol, stay in the system until they receive service from a group of servers. Such queues are typically characterized by specifying a) the stochastic process governing the arrival of customers, b) distribution of number of customers in each arrival, c) the stochastic process governing departure of customers, and d) the number of servers. When the gene expression model in Fig 1 is expressed in the language of queueing theory, individual mRNAs/proteins are the analogs of customers in queueing models. The production of mRNAs/proteins in bursts corresponds to the arrival of customers in batches. Just as the customers leave the queue after receiving service, mRNAs/proteins exit the system upon degradation. Thus the waiting-time distribution for mRNA/protein decay is the analog of service time distribution for customers in queueing models. For the model in Fig 1, their decay time distribution is the exponential distribution. Also, since mRNAs/proteins are degraded independently of each other, the corresponding number of servers in queueing models is ∞ (which ensures that presence of a customer in the system does not affect the service time of any other customer in the system).

Based on the above mapping, the queueing system corresponding to the model outlined in Fig 1 is the *GI* ^*X*^/*M*/∞ system [43, 48]. In this model, the symbol *G* refers to a general waiting-time distribution for the arrival process, *I* ^*X*^ denotes customers arriving in batches of independently distributed random sizes *X*, *M* stands for Markovian (i.e. exponential) service-time distribution for customers and ‘∞’ stands for infinite servers.

For the *GI* ^*X*^/*M*/∞ model, exact results for iteratively obtaining the moments of the steady-state distribution of the number of customers have been derived [48]. Using these results, explicit expressions for the first four moments of the steady-state distribution are provided in the Supplementary S1 Text. Applying the mapping discussed above, these results can be translated into exact expressions for the moments of mRNA/protein steady-state distributions, as discussed below.

Let us first examine the expressions for steady-state means of mRNAs, ⟨*m*_*s*_⟩, and proteins, ⟨*p*_*s*_⟩, which are given by

$$\left\langle m_{s}\right\rangle =\frac{k_{b}}{\mu_{m}}\left\langle m_{b}\right\rangle ,\;\left\langle p_{s}\right\rangle =\frac{k_{b}}{\mu_{p}}b,$$ (2)

where *k*_*b*_ stands for the mean arrival rate of mRNA bursts and *b* = ⟨*m*_*b*_⟩⟨*p*_*b*_⟩ is the mean of the protein burst distribution (including contributions from all the mRNAs). Although Eq (2) has been derived by assuming that the arrival of mRNAs/proteins is a renewal process, it is valid for arbitrary arrival processes. This is because Eq (2) is a direct consequence of Little’s Law [47, 53] which is valid for general arrival processes.

The above equations, Eq (2), can be used to determine the mean transcriptional burst size, provided the protein burst distribution can be measured experimentally. To see this, we note that dividing the expressions for the mean mRNA and protein levels leads to

$$\frac{b}{\left\langle m_{b}\right\rangle }=\frac{\mu_{p}}{\mu_{m}}\frac{\left\langle p_{s}\right\rangle }{\left\langle m_{s}\right\rangle }.$$ (3)

Since the steady-state means ⟨*m*_*s*_⟩ and ⟨*p*_*s*_⟩ as well as the degradation rates *μ*_*m*_ and *μ*_*p*_ are parameters that can be measured experimentally, the above equation implies that the ratio *b*/⟨*m*_*b*_⟩ can be determined experimentally. Given *b*/⟨*m*_*b*_⟩ = *k*_*p*_/*μ*_*m*_, this implies that the mean protein production rate *k*_*p*_ can also be determined experimentally. This is an important result since it provides an approach for determining the mean protein production rate *k*_*p*_ that is valid for arbitrary arrival processes for mRNAs. Furthermore, the above equation implies that, if the mean of protein burst distribution *b* can be measured [36], then the mean transcriptional burst size ⟨*m*_*b*_⟩ can also be determined. Thus, if we have measurements for mean mRNA and protein numbers and also the mean of protein burst distribution, then these measurements can be used to determine the degree of transcriptional bursting ⟨*m*_*b*_⟩ as well as the parameters ⟨*p*_*b*_⟩ and *k*_*p*_. It is noteworthy that this procedure for estimating the burst parameters is valid for arbitrary stochastic processes corresponding to mRNA transcription.

We next turn to expressions for higher moments of mRNA and protein steady-state distributions. The noise in mRNA steady-state distributions is given by

$$\begin{array}{ccc} \frac{\sigma_{m_{s}}^{2}}{{\left\langle m_{s}\right\rangle }^{2}} & = & \frac{1}{\left\langle m_{s}\right\rangle }+\frac{\mu_{m}}{k_{b}}+\frac{\mu_{m}}{2k_{b}}[K_{g}\left(\mu_{m}\right)-1+\frac{\sigma_{m_{b}}^{2}}{{\left\langle m_{b}\right\rangle }^{2}}\\ -(1+\frac{1}{\left\langle m_{b}\right\rangle })], \end{array}$$ (4)

where $\sigma_{m_{b}}^{2}$ is the variance of mRNA burst distribution and *K*_*g*_(*μ*_*m*_) is the so-called gestation factor,

$$K_{g}\left(\mu_{m}\right)=1+2[\frac{f_{L}\left(\mu_{m}\right)}{1-f_{L}\left(\mu_{m}\right)}-\frac{k_{b}}{\mu_{m}}],$$ (5)

with *f*_*L*_(*s*) denoting the Laplace transform of arrival time distribution of mRNA bursts. The function *K*_*g*_(*μ*_*m*_) encodes information about the arrival process. Specifically, we note that for Poisson arrivals, we have *K*_*g*_(*μ*_*m*_) = 1.

For proteins (in the burst limit *μ*_*m*_ ≫ *μ*_*p*_), we obtain [43]

$$\begin{array}{cc} \frac{\sigma_{p_{s}}^{2}}{{\langle p_{s}\rangle }^{2}} & =\frac{1}{\langle p_{s}\rangle }+\frac{\mu_{p}}{k_{b}}+\frac{\mu_{p}}{2k_{b}}[K_{g}(\mu_{p})-1+\;\frac{\sigma_{m_{b}}^{2}}{{\langle m_{b}\rangle }^{2}}\\ & -\left(1-\frac{1}{\langle m_{b}\rangle }\right)\;+\left(\frac{\sigma_{p_{b}}^{2}}{{\langle p_{b}\rangle }^{2}}\;-\left(1+\frac{1}{\langle p_{b}\rangle }\right)\right)\frac{1}{\langle m_{b}\rangle }] \end{array}$$ (6)

where *K*_*g*_(*μ*_*p*_) is given by Eq (5) and $\sigma_{p_{b}}^{2}$ is the variance of protein burst distribution produced by a single mRNA. The expression for protein noise is composed of the noise term for the basic two-stage model of gene expression [40] and additive noise contributions due to: a) deviations from exponential waiting-time distribution for the arrival process, b) deviations from conditional geometric distributions for mRNA burst distributions and c) deviations from geometric distributions for protein burst distributions. For both mRNAs and proteins, the noise in steady-state distributions depends on all the moments of the burst arrival time distribution through the term *K*_*g*_. Therefore, arrival processes corresponding to different kinetic schemes for transcription will make different contributions to the overall noise, even if they have identical means and variances for the the burst arrival time distribution.

We note from Eq (4) that, for Poisson arrivals, i.e. *K*_*g*_ = 1, and geometrically distributed burst, i.e. $\sigma_{m_{b}}^{2}=\langle m_{b}\rangle (\langle m_{b}\rangle -1$), the equations for the noise and mean have only two unknown burst parameters, *k*_*b*_ and ⟨*m*_*b*_⟩. In this case, experimental measurements of the first two moments of the steady-state distribution are sufficient to estimate the burst parameters, as has been done in multiple studies. However, when the arrival process is non-Poisson or if the burst distribution deviates from a geometric distribution, measurements of the first two steady-state moments are not sufficient for estimating the burst parameters. This observation motivates the need for analytical expressions for the higher moments which we turn to next.

We now derive analytical expressions for the third moment, specifically the skewness parameter. For mRNAs, the exact expression for skewness *γ*_*m*_*s*__ is given by

$$\begin{array}{ccc} \frac{\gamma_{m_{s}}\sigma_{m_{s}}^{3}}{m_{s}} & = & 1+\left\langle m_{s}\right\rangle \left\langle m_{b}\right\rangle K_{1}\left(\mu_{m}\right)+2{\left\langle m_{b}\right\rangle }^{2}K_{2}\left(\mu_{m},\left\langle m_{b}\right\rangle \right)\\ & + & (\sigma_{m_{b}}^{2}+{\left\langle m_{b}\right\rangle }^{2}-\left\langle m_{b}\right\rangle )K_{3}\left(\mu_{m},\left\langle m_{b}\right\rangle \right)\\ & + & \frac{\left\langle m_{b}\left(m_{b}-1\right)\left(m_{b}-2\right)\right\rangle }{3\langle m_{b}\rangle }, \end{array}$$ (7)

where we have defined

$$\begin{array}{ccc} K_{1}\left(\mu_{m}\right) & = & K_{g}\left(2\mu_{m}\right)-K_{g}\left(\mu_{m}\right),\\ K_{2}\left(\mu_{m},\left\langle m_{b}\right\rangle \right) & = & \frac{K_{g}\left(\mu_{m}\right)-1}{4}(\frac{3}{\left\langle m_{b}\right\rangle }+K_{g}\left(2\mu_{m}\right)-1),\\ K_{3}\left(\mu_{m},\left\langle m_{b}\right\rangle \right) & = & \frac{3}{2\langle m_{b}\rangle }+\frac{K_{g}\left(\mu_{m}\right)+K_{g}\left(2\mu_{m}\right)}{2}-1. \end{array}$$ (8)

For proteins, we obtain in the burst-limit (*μ*_*m*_ ≫ *μ*_*p*_),

$$\begin{array}{ccc} \frac{\gamma_{p_{s}}\sigma_{p_{s}}^{3}}{p_{s}} & = & 1+{\left(A_{1}^{p}\right)}^{2}[\frac{\left\langle p_{s}\right\rangle }{b}K_{1}\left(\mu_{p}\right)+2K_{2}\left(\mu_{p},A_{1}^{p}\right)]\\ & + & A_{2}^{p}K_{3}\left(\mu_{p},A_{1}^{p}\right)+\frac{A_{3}^{p}}{3A_{1}^{p}}, \end{array}$$ (9)

where the functions $K_{1}$, $K_{2}$, $K_{2}$ are given by Eq (8), $A_{k}^{p}$ is defined by $A_{k}^{p}=d^{k}A^{p}\left(z\right)/dz^{k}{|}_{z=1}$ and, using Eq (1) [54], we obtain the parameters $A_{k}^{p}$ as:

$$\begin{array}{ccc} A_{1}^{p} & = & \langle m_{b}\rangle \langle p_{b}\rangle ,\\ A_{2}^{p} & = & \langle m_{b}\rangle \left(\sigma_{p_{b}}^{2}-\langle p_{b}\rangle \right)+\left(\sigma_{m_{b}}^{2}+{\langle m_{b}\rangle }^{2}\right){\langle p_{b}\rangle }^{2},\\ A_{3}^{p} & = & {\langle p_{b}\rangle }^{3}\langle m_{b}(m_{b}-1)(m_{b}-2)\rangle +3\langle m_{b}(m_{b}-1)\rangle \langle p_{b}\rangle \;\\ & & \langle p_{b}(p_{b}-1)\rangle +\langle m_{b}\rangle \;\langle p_{b}(p_{b}-1)(p_{b}-2)\rangle . \end{array}$$ (10)

Similarly, expressions for higher order moments of protein and mRNA steady-state distributions can be obtained iteratively. The corresponding expressions for the kurtosis are provided in the S1 Text.

The analytical results derived above for proteins are exact in the burst limit, which assumes that proteins are produced instantaneously from all the mRNAs in a burst. Going beyond the burst limit (i.e. not limited to *μ*_*m*_ ≫ *μ*_*p*_), exact results for the higher moments of the protein steady-state distribution will, in general, depend on the details of the kinetic scheme for gene expression. However, we can derive approximate analytical expressions for general schemes by requiring that: a) the results reduce to the exact results in the burst limit and b) they match the exact results for the two-stage model of gene expression. For the two-stage model, exact results for the first four moments have been derived by Bokes et. al [55]. Comparing these exact results with our results derived in the burst limit, we observe that results of [55] can be reproduced by a suitable scaling of the burst-size parameters $A_{k}^{p}$. For example, the exact expression for the noise is obtained by the following scaling [43]:

$$(\frac{\sigma_{p_{s}}^{2}}{{\left\langle p_{s}\right\rangle }^{2}}-\frac{1}{\left\langle p_{s}\right\rangle })\to (\frac{\sigma_{p_{s}}^{2}}{{\left\langle p_{s}\right\rangle }^{2}}-\frac{1}{\left\langle p_{s}\right\rangle })\frac{1}{1+\frac{\mu_{p}}{\mu_{m}}}.$$ (11)

Similarly, for the expression for skewness, the parameters $A_{2}^{p}$ and $A_{3}^{p}$ are scaled as:

$$A_{2}^{p}\to A_{2}^{p}\frac{1}{1+\frac{\mu_{p}}{\mu_{m}}}\;\text{and}\;A_{3}^{p}\to A_{3}^{p}\frac{1}{\left(1+\frac{\mu_{p}}{\mu_{m}}\right)\left(1+2\frac{\mu_{p}}{\mu_{m}}\right)}.$$ (12)

**Fig 2:**
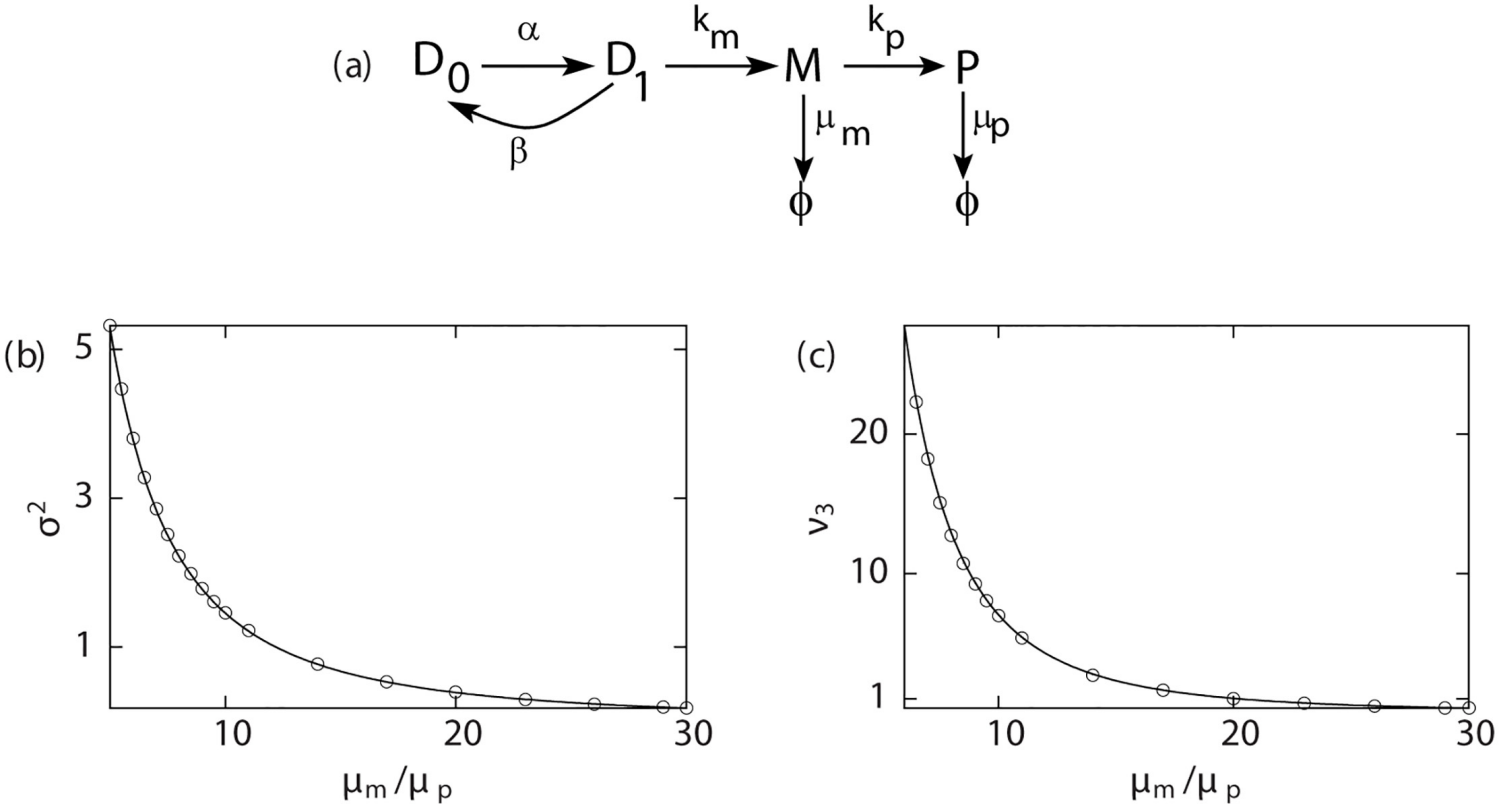
Steady state moments for proteins. (a) Kinetic scheme for the two-state random telegraph model. For this model, steady state variance (scaled by 10^−5^) and third central moment *ν*_3_ (scaled by 10^−6^) of proteins as a function of *μ*_*m*_/*μ*_*p*_ are plotted in (b) and (c) respectively: lines represent analytic estimates and points correspond to the simulation results. Parameters are: *α* = 0.5, *β* = 0.25, *k*_*m*_ = 2, ⟨*m*_*b*_⟩ = 5, *k*_*p*_ = 0.5.

As shown in Fig 2 (for the random telegraph model) the analytical expressions using this approach are in good agreement with results from simulations [56].

It is noteworthy that the results derived are valid for a general class of kinetic schemes of gene expression. For a specific kinetic scheme, we can determine the corresponding waiting-time distribution for the arrival process and the burst distributions for mRNA and proteins. Substituting these results in the equations derived leads to the corresponding expressions for the moments of the steady-state distribution. The results obtained can thus provide insight into how specific kinetic schemes of gene expression (e.g. combining promoter-based regulation and post-transcriptional regulation) can be used to impact the noise and higher moments of steady-state distributions.

### Estimation of burst parameters

The results derived for the steady-state moments indicate that, if the burst arrival process is not a Poisson process, then it is no longer accurate to estimate burst parameters based on measurements of mean and variance only, as has been done in previous studies [13]. In the following, we present approaches for estimating burst parameters in the general case.

**Fig 3:**
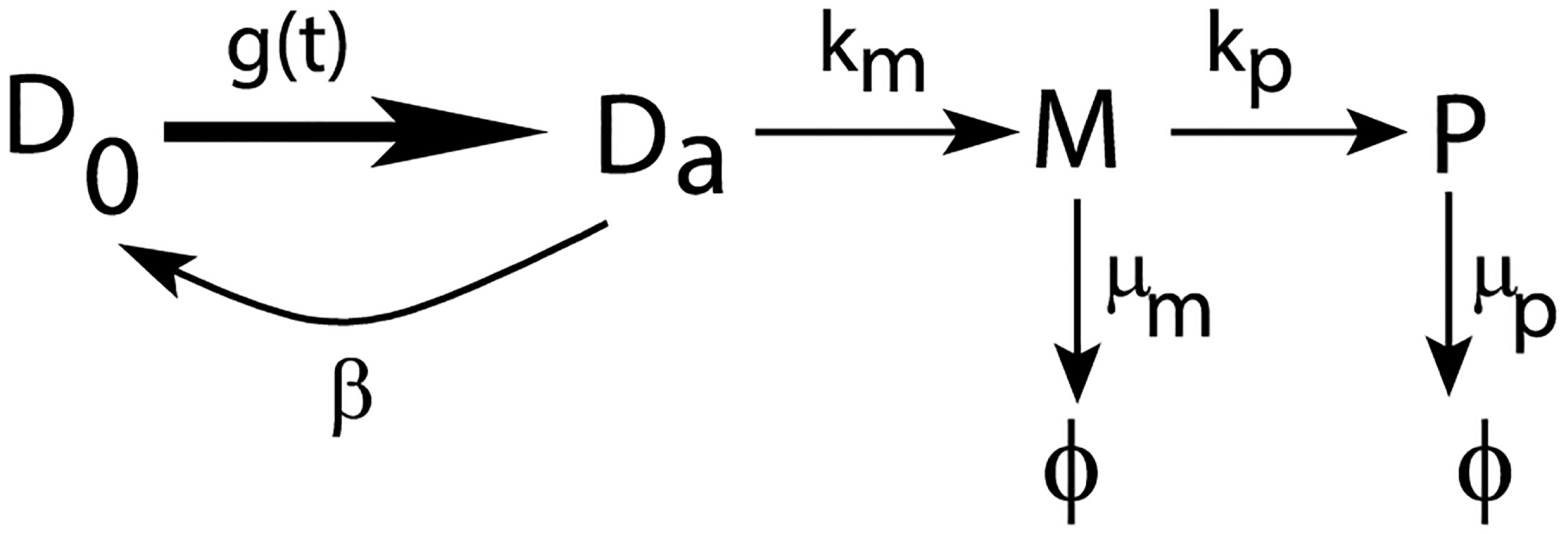
Schematic representation of the general kinetic scheme with promoter switching. Thick line from inactive state *D*_0_ to active state *D*_*a*_ represents a general kinetic scheme with *g*(*t*) as the waiting-time distribution for the promoter to switch to the ON state.

We begin by considering the general kinetic scheme shown in Fig 3. This form for the kinetic scheme is supported by recent experiments in mammalian cells which suggest the presence of multiple rate-limiting steps between transition of the promoter from OFF to ON state [45, 57]. However, as observed in these experiments, a promoter in the ON state switches to the OFF state by a single rate-limiting step. We model the promoter switching from OFF to ON state by a general waiting-time distribution, *g*(*t*). The switching rate from ON to OFF state is given by *β*.

#### Burst parameters from the sequence-size function

To extract burst parameters for the general scheme considered above, we first note that bursts are generated due to the interplay of two time scales, one that corresponds to production of mRNAs (when the gene is active) while the other one corresponds to the waiting-time between production events (when the gene is in inactive state). For bursty gene expression, we expect a clear separation of time-scales between the characteristic time periods for these two cases. Following [41], it is convenient to define a sequence-size function,

$$\phi \left(\tau \right)=\frac{1}{1-\int_{0}^{\tau }f\left(t\right)dt},$$ (13)

where *f*(*t*) is the waiting-time distribution for the arrival of a *single* mRNA starting with the promoter in the ON state. For a fixed *τ*, the sequence-size function can be used to categorize time intervals larger than *τ* as separating bursts. Correspondingly, the term $1-\int_{0}^{\tau }f\left(t\right)dt$ represents the fraction of all mRNA arrivals that correspond to the arrivals produced in a single burst; thus *ϕ* provides the corresponding mean burst size. For bursty gene expression with a separation of time-scales, for a specific choice of *τ* = *τ*_*x*_, the sequence-size function can be related to the actual mean burst size. If *f*(*t*) can be measured, then determination of *τ*_*x*_ can result in accurate estimates of the burst parameters such as mean burst size and frequency. In the following, we discuss how to determine *τ*_*x*_ for the general class of arrival processes considered in Fig 3.

The key insight is based on the observation that, due to the separation of time scales within bursts and between consecutive bursts, determination of *τ*_*x*_ can be done by using a simple two-state model as shown in Fig 2a. Even though the actual waiting time distribution between bursts (*g*(*t*)) may differ from the exponential distribution for the two-state model, the short-time behavior of the sequence-size function will be indistinguishable between the two cases (given separation of time-scales). If *τ*_*x*_ can be connected to the short-time behavior, then analytical expressions for the sequence-size function *ϕ*(*τ*) for the two-state model can be used to estimate *τ*_*x*_ and thereby the mean burst size.

For the two-state model, we find that burst size can be determined using a specific *τ*_*x*_, which corresponds to an inflexion point where the curvature of *ϕ*(*τ*) changes its sign. Specifically, for the two-state model, we obtain *f*(*t*) by taking inverse Laplace transform of *f*(*s*) given by Eq (23). In the burst-limit, i.e., *α*/*β* → 0, we find that the sequence-size function, using Eq (13), is given by

$$\begin{array}{c} \phi \left(\tau \right)=\frac{(k_{m}+\beta )e^{\tau (k_{m}+\beta )}}{k_{m}+\beta e^{\tau (k_{m}+\beta )}}, \end{array}$$ (14)

and the value of *τ* at which *ϕ*(*τ*) exhibits inflexion is

$$\tau_{x}=\frac{1}{k_{m}+\beta }\ln\frac{k_{m}}{\beta },\;k_{m}>\beta .$$ (15)

The sequence size function *ϕ*(*τ*) at this point (*τ* = *τ*_*x*_) is given by:

$$\phi \left(\tau_{x}\right)=\frac{1}{2}(1+\frac{k_{m}}{\beta })=\frac{1}{2}(1+\langle m_{b}\rangle ),$$ (16)

Thus, the procedure for determination of the mean burst size (1 + ⟨*m*_*b*_⟩), given *f*(*t*), is as follows:

1. Obtain the sequence-size function *ϕ*(*τ*) from *f*(*t*). For bursty synthesis, *ϕ*(*τ*) will have an inflexion point.
2. The mean burst size (1 + ⟨*m*_*b*_⟩) is simply twice the value of the the sequence-size function *ϕ*(*τ*) at the inflexion point, *τ*_*x*_.

This approach has been validated using stochastic simulations for multiple promoter models with correspondingly complex waiting-time distributions between bursts.

#### Estimation of *f*(*t*) from steady-state moments

The procedure outlined in the previous section assumes that the waiting-time distribution *f*(*t*) can be determined. However, this can be challenging experimentally, thus it is desirable to develop approaches for estimating *f*(*t*) based on measurements of steady-state distributions.

To proceed in this direction, let us first obtain a relation connecting the two waiting-time distributions *f*(*t*) (for single mRNA arrival) and *g*(*t*) (for burst arrival). In Fig 3, we note that when the promoter is in the active state, *D*_*a*_, it can make multiple trips to *D*_0_ before producing mRNA. Whenever gene is in *D*_*a*_ state, it can either create mRNA or can switch back to *D*_0_ state. Gene in *D*_*a*_ state can produce mRNA either in a single step, i.e., without switching back to *D*_0_ state, or by making multiple trips to *D*_0_ before producing mRNA. Denoting the number of trips made before producing mRNA by *q*, we obtain that the Laplace transform of the waiting-time distribution *f*(*t*) is given by

$$f_{L}(s)=\frac{k_{m}}{\beta +k_{m}}\;\sum_{q=0}^{\infty }{\left(\frac{\beta }{\beta +k_{m}}\right)}^{q}{\left[g_{L}(s)\right]}^{q}{\left(\frac{k_{m}+\beta }{k_{m}+\beta +s}\right)}^{q+1},$$ (17)

which leads to:

$$f_{L}\left(s\right)=\frac{k_{m}}{k_{m}+s+[1-g_{L}\left(s\right)]\beta }.$$ (18)

In order to determine *f*_*L*_(*s*), we will assume a specific functional form for *g*_*L*_(*s*). We consider that *g*_*L*_(*s*) is given by the following rational function,

$$\begin{array}{c} g_{L}\left(s\right)\equiv g_{n}^{m}\left(s\right)=\frac{1+a_{1}s+a_{2}s^{2}\cdots a_{m}s^{m}}{1+b_{1}s+b_{2}s^{2}\cdots b_{n}s^{n}},\;n>m. \end{array}$$ (19)

This form for the Laplace transform of the waiting-time distribution is consistent with known waiting-time distributions for phase-type processes [54] and thus is valid quite generally.

Once we have an explicit form for *f*_*L*_(*s*), the next step is to determine the parameters, *k*_*m*_, *β*, *a*_1_…*a*_*m*_, and *b*_1_…*b*_*n*_. Thus, in general, we need *m*+*n*+2 measurements to estimate these parameters if we use $g\left(s\right)=g_{n}^{m}\left(s\right)$. The simplest case, $g_{L}\left(s\right)=g_{1}^{0}\left(s\right)$, implies the presence of one kinetic step from inactive state to active state, with rate 1/*b*_1_, and so it corresponds to the standard two-state random telegraph model. For this simple kinetic scheme, we can find the parameters, *k*_*m*_, *β*, and *b*_1_, and hence *f*_*L*_(*s*) and the sequence size function by using three measurements associated with either mRNAs or proteins.

**Fig 4:**
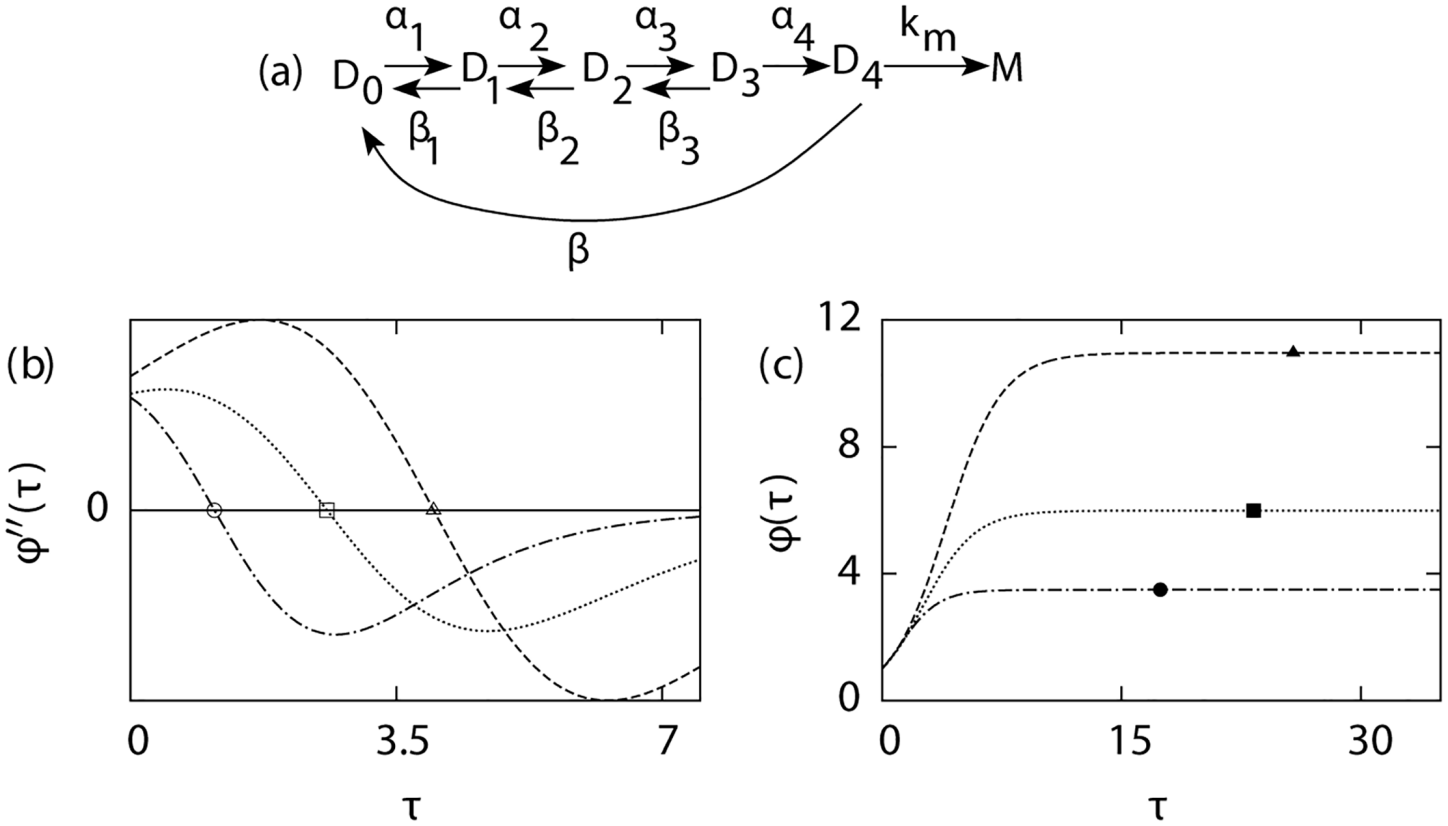
Estimation of mean burst size from sequence size function *ϕ*(*τ*). For the transcriptional scheme shown in (a), the variations of *ϕ*″(*τ*) and *ϕ*(*τ*) as a function of time *τ* (scaled by 10^3^) are shown in (b) and (c) respectively. The three lines correspond to three different values of *β*, 50 (dashed line), 100 (dotted line) and 200 (dashed-dotted line), while keeping *k*_*m*_ = 500: Exact burst size for these three cases are 11, 6 and 3.5, respectively. Estimated mean burst size has been indicated by filled symbols and the inflexion points in the sequence size function are shown by empty symbols. Other parameters: *α*_1_ = 1,*α*_2_ = 0.5,*α*_3_ = 0.25,*α*_4_ = 0.75,*β*_1_ = 0.1,*β*_2_ = 0.2,*β*_3_ = 0.5.

The form, $g_{L}\left(s\right)=g_{1}^{0}\left(s\right)$, is exact for the two-state random telegraph model. Using the expressions obtained for the first four steady-state moments, we can derive an analytic condition that determines whether the underlying mechanism can be represented by $g_{1}^{0}\left(s\right)$ (see Supplementary S2 Text). However, if the arrival process is complex and involves multiple rate-limiting steps, then $g_{1}^{0}\left(s\right)$ will not be an accurate representation of the underlying kinetic process. In such cases, we need to use *g*_*L*_(*s*) of higher order. The next step in this iterative process is to take $g_{L}\left(s\right)=g_{2}^{0}\left(s\right)$. This form of *g*_*L*_(*s*) is valid if there are only two rate-limiting steps in the promoter transition from OFF to ON state. For kinetic schemes that involve more than two steps, it will serve as an approximate reduced representation. Interestingly, it turns out that even if $g_{L}\left(s\right)=g_{2}^{0}\left(s\right)$ is not a correct representation of the underlying kinetic process, this reduced representation works very well as far as estimating burst size is concerned. In Fig 4, we have illustrated the effectiveness of this approach for a complex kinetic scheme for the promoter transition from OFF to ON state. The figure also illustrates the effectiveness of the approach outlined in the previous subsection for determining the mean burst size using the sequence-size function *ϕ*(*τ*).

While the reduced representation, $g_{L}\left(s\right)=g_{2}^{0}\left(s\right)$, works reasonably well for estimating burst size, with additional data, it is possible to extend the process further. The iterative procedure we propose is as follows:

1. Start with the simplest form $g_{1}^{0}\left(s\right)$ and use three moments associated with either mRNA or proteins (or both) to find *f*_*L*_(*s*) as discussed above. Then this *f*_*L*_(*s*) can be used to get analytic predictions for higher moments [48].
2. If these analytic predictions are consistent with the corresponding experimental observations then $g_{1}^{0}\left(s\right)$ provides a reasonable representation of the underlying kinetic scheme, else a representation using more complex kinetic schemes is required.
3. To address more complex kinetic schemes, we iteratively change *g*_*L*_(*s*) from $g_{1}^{0}\left(s\right)$ to $g_{2}^{0}\left(s\right)$…and so on, and iterate the steps outlined to determine the underlying *f*_*L*_(*s*). However, we note that for uncovering more complex kinetic scheme we need additional measurements to estimate *f*_*L*_(*s*). If moment measurements are possible at different mRNA/protein degradation rates, then these additional measurements can be used to estimate *f*_*L*_(*s*) and hence the corresponding mean transcriptional burst size.

#### Effect of extrinsic noise on burst estimation

The burst estimation approach discussed in the preceding section assumes that the dominant contribution comes from intrinsic sources of fluctuations. However, extrinsic noise [1, 12], e.g. arising from different concentration of cellular components such as RNA polymerase, can also contribute significantly to the observed variations. It is thus of interest to examine how the proposed burst parameter estimation procedure works if we also consider sources of extrinsic noise.

**Fig 5:**
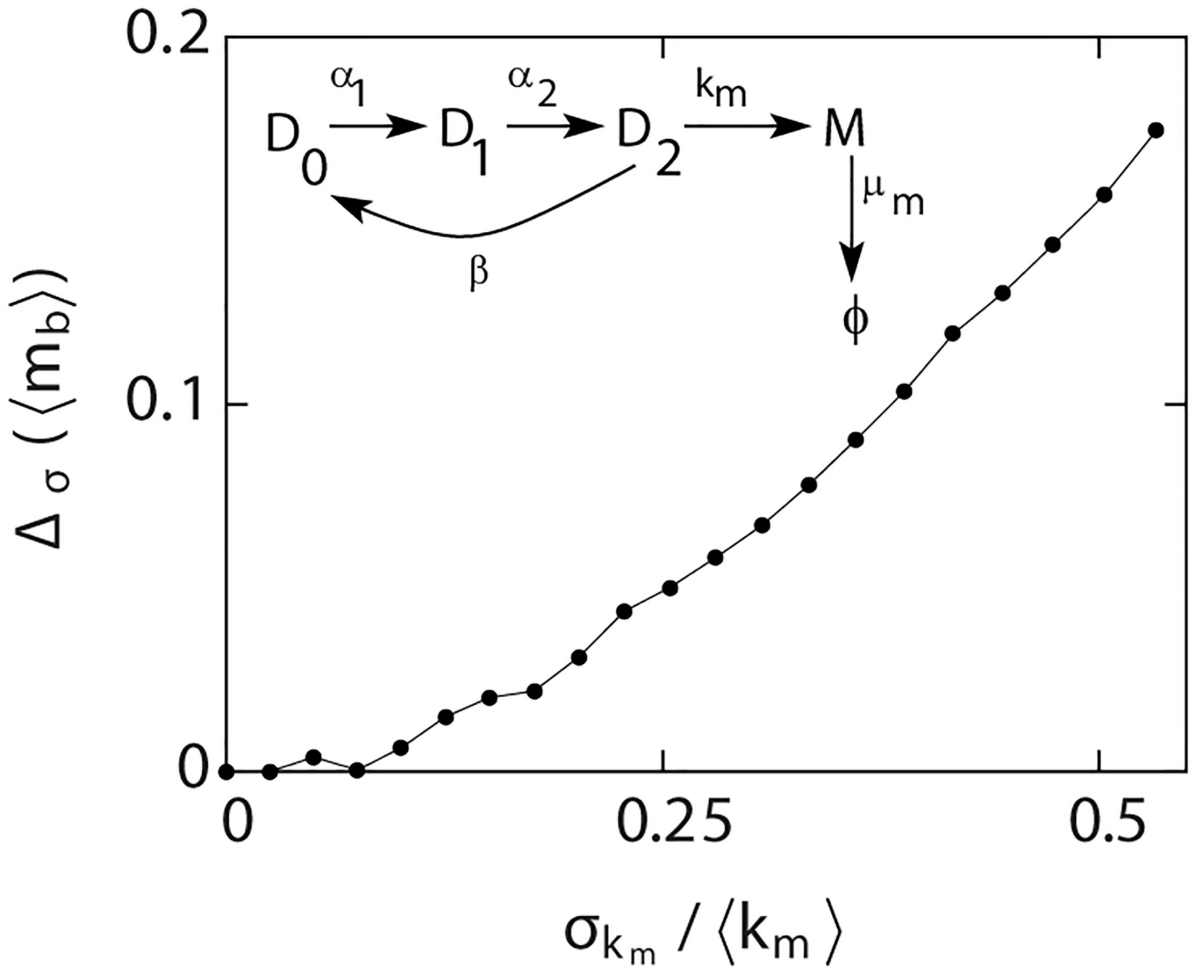
Effects of extrinsic noise on burst estimation. For the transcriptional scheme shown in the inset, the relative error Δ_*σ*_(⟨*m*_*b*_⟩) = (⟨*m*_*b*_⟩_0_−⟨*m*_*b*_⟩_*σ*_)/⟨*m*_*b*_⟩_0_ is plotted. Parameters as *α*_1_ = 1, *α*_2_ = 0.5, *β* = 50, ⟨*k*_*m*_⟩ = 500 and *μ*_*m*_ = 1.

To explore the effects of such fluctuations, we consider the model shown in Fig 5. In this kinetic scheme, the activation of gene from OFF to ON state involves two sequential steps, with rates *α*_1_ and *α*_2_. To include extrinsic fluctuations in the model, we consider that the rate of transcription *k*_*m*_ is a Log-normally distributed random variable with mean ⟨*k*_*m*_⟩ and standard deviation *σ*_*k*_*m*__. For a given value of *σ*_*k*_*m*__, we determine the mean burst size following the procedure outlined above: i.e. by taking $g\left(s\right)=g_{2}^{0}\left(s\right)$ and then using the simulation values for the first four steady-state moments of mRNAs to estimate the unknown parameters (*b*_1_,*b*_2_,*k*_*m*_,*β*), and hence the burst size. By varying *σ*_*k*_*m*__ we study how the estimated burst size ⟨*m*_*b*_⟩_*σ*_ deviates from the one without extrinsic noise, ⟨*m*_*b*_⟩_0_. As can be seen in Fig 5, for smaller values of *σ*_*k*_*m*__, the estimated burst size ⟨*m*_*b*_⟩_*σ*_ is reasonably close to ⟨*m*_*b*_⟩_0_, however, as expected, ⟨*m*_*b*_⟩_*σ*_ shows monotonic deviations from ⟨*m*_*b*_⟩_0_ for larger values of *σ*_*k*_*m*__.

### Signatures for non-Poisson arrivals

The analytical expressions derived for the steady-state moments for mRNAs and proteins can also be used to make inferences about the burst arrival process based on steady-state measurements. Since multiple studies assume that the burst arrival process is characterized by an exponential waiting-time distribution, it would be useful to determine if this assumption is invalid using measurements of steady-state distributions. As shown below, we can obtain conditions for the same using the results derived for higher moments.

In the following, we will first focus on the cases that the mRNA burst distribution is conditional geometric and the protein burst distribution is geometric, which is consistent with multiple experimental observations. As discussed, choosing the conditional geometric distribution for mRNAs allows us to consider both single mRNA arrivals and geometric mRNA bursts in one framework. Since experiments can provide measurements of both mRNA and protein steady-state distributions, it is useful to have conditions for the arrival process using either mRNA data or protein data or both mRNA and protein data. Based on these three possibilities, we present three different conditions in the following.

#### Using moments of mRNA steady-state distributions

Let us first consider the case where we have only measurements of the mRNA steady-state distribution. We note that for Poisson arrivals *K*_*g*_(*μ*_*m*_) = *K*_*g*_(2*μ*_*m*_) = 1, and using the expressions for mean and noise from Eqs (2) and (4) we get, *F*_*m*_ = ⟨*m*_*b*_⟩, where $F_{m}=\sigma_{m_{s}}^{2}/\langle m_{s}\rangle$ is the mRNA Fano factor. Further, using this in the equation for skewness, Eq (7), we derive the following condition that must be satisfied if the arrival of mRNA bursts is a Poisson process:

$$D_{m}\equiv \frac{\gamma_{m_{s}}\sigma_{m_{s}}^{3}}{\left\langle m_{s}\right\rangle [3\left(F_{m}-1\right)\{1+\frac{2}{3}\left(F_{m}-1\right)\}+1]}-1=0.$$ (20)

Thus $D_{m}\neq 0$ is a signature of non-Poisson arrival processes. Since the above prescription is based on experimentally measurable quantities such as $\langle m_{s}\rangle ,\sigma_{m_{s}}^{2},\gamma_{m_{s}}$ and *μ*_*m*_, it can be used to determine if the assumption of a Poisson arrival process is invalid.

#### Using moments of protein steady-state distributions

We next consider the case where we have access to only the protein steady-state distribution. The steps followed are similar to those outlined for the mRNA case. For Poisson arrivals, *K*_*g*_(*μ*_*p*_) = 1, and using Eqs (2) and (11) we get

$$\begin{array}{c} b=\left(F_{p}-1\right)(1+\frac{\mu_{p}}{\mu_{m}}), \end{array}$$

where $F_{p}=\sigma_{p_{s}}^{2}/\langle p_{s}\rangle$ is the protein Fano factor. Substituting this in the expression for protein skewness, Eq (9) with the scaled $A_{2}^{p}$ and $A_{3}^{p}$ given by Eq (12), we arrive at the following condition for Poisson arrivals.

$$\begin{array}{ccc} D_{p} & \equiv & \frac{\gamma_{p_{s}}\sigma_{p_{s}}^{3}}{\left\langle p_{s}\right\rangle [3\left(F_{p}-1\right)\{1+\frac{2}{3}(\frac{\mu_{m}+\mu_{p}}{\mu_{m}+2\mu_{p}})\left(F_{p}-1\right)\}+1]}-1\\ & = & 0 \end{array}$$ (21)

Again, non-zero value of $D_{p}$ is a signature of non-Poisson arrivals.

#### Using both mRNA and protein steady-state distributions

Finally, if we have both mRNA and protein steady-state distribution measurements available, then the condition for Poisson arrivals can be obtained by combining measurements of second moments of mRNA and protein distributions as follows: Using Eqs (2),(4) and (11), we get,

$$\begin{array}{ccc} D_{mp} & \equiv & \frac{F_{m}}{\left\langle m_{s}\right\rangle \mu_{m}}-\frac{\left(\mu_{m}+\mu_{p}\right)\left(F_{p}-1\right)}{\mu_{m}\left\langle p_{s}\right\rangle \mu_{p}}\\ & = & \frac{1}{2k_{b}}[K_{g}\left(\mu_{m}\right)-K_{g}\left(\mu_{p}\right)], \end{array}$$ (22)

which vanishes for Poisson arrival of mRNA bursts. Thus non-zero values of $D_{mp}$ indicate non-Poisson arrival of mRNA bursts. Interestingly, for this condition there is no need to assume that the mRNA burst distribution is geometric. That is, the condition holds true for arbitrary mRNA burst distributions. Also, the condition does not require measurement of third moments.

#### Signatures for a simple kinetic scheme

To illustrate the prescription derived for determining non-Poisson arrival processes, we consider a specific kinetic scheme, Fig 2a. For this kinetic scheme, the mRNA arrival time distribution in the Laplace domain is given by (Eq (S3–9) Supplementary S3 Text)

$$f_{L}\left(s\right)=\frac{k_{m}(\alpha +s)}{k_{m}(\alpha +s)+s(\alpha +\beta +s)}.$$ (23)

Using this in Eq (5) we find the gestation factor, *K*_*g*_, and hence the mean, Fano factor and skewness for both mRNAs and proteins. Finally, we derive exact analytic expressions for $D_{m}$, $D_{p}$ and $D_{mp}$ from Eqs (20), (21) and (22) respectively. The expression for $D_{m}$ reads

$$D_{m}=\frac{2k_{m}\beta \left(1-\left\langle m_{b}\right\rangle \right)\theta (1+\frac{\left(1+\alpha \right)\left(\alpha +\beta \right)\left\langle m_{b}\right\rangle k_{m}}{\theta (\left\langle m_{b}\right\rangle -1)})}{\left(2+\alpha +\beta \right)\left(\theta +\beta k_{m}\right)\left(\left(2\left\langle m_{b}\right\rangle -1\right)\theta +2k_{m}\left\langle m_{b}\right\rangle \beta \right)},$$ (24)

where

θ=(α+β)(α+β+1), (25)

and we have set *μ*_*m*_ = 1 for simplicity. As expected, we note that $D_{m}$ vanishes for the Poisson arrival processes, i.e., either when *β* is zero, or when the switching rates *α* and *β* are very large compared to the rate of transcription, *k*_*m*_. The general expression for $D_{p}$ is complicated. However, to gain insight about the arrival process, we can write down a simpler expression for $D_{p}$ in the burst limit, *μ*_*m*_ = 1 ≫ *μ*_*p*_:

$$D_{p}=-\frac{2{\left\langle m_{b}\right\rangle }^{2}k_{m}^{2}k_{p}^{2}\alpha \beta }{{\left(\alpha +\beta \right)}^{4}+3\left\langle m_{b}\right\rangle k_{p}{\left(\alpha +\beta \right)}^{2}\psi +2{\left\langle m_{b}\right\rangle }^{2}k_{p}^{2}\psi^{2}},$$ (26)

where

$$\psi =k_{m}\beta +{\left(\alpha +\beta \right)}^{2}.$$ (27)

**Fig 6:**
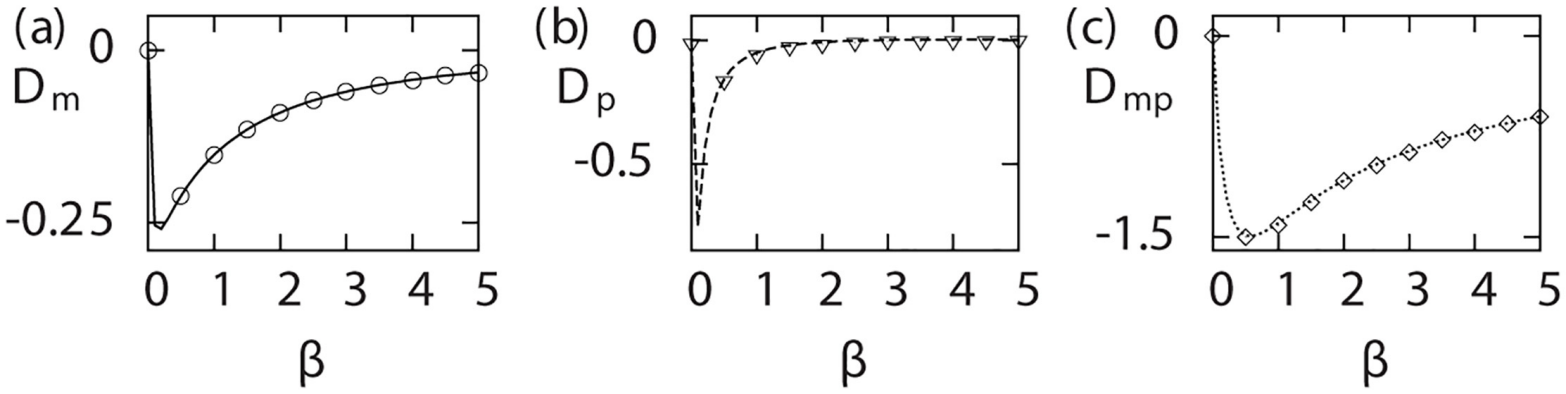
Signatures for non-Poisson arrival. The quantities $D_{m}$, $D_{p}$ and $D_{mp}$ are plotted for the model shown in Fig 2a as a function of *off* rate *β*. Analytic estimates are shown by lines whereas points correspond to the simulation results with parameters: *α* = 0.25, *k*_*m*_ = 2, ⟨*m*_*b*_⟩ = 5, *k*_*p*_ = 0.5, *μ*_*m*_ = 1, *μ*_*p*_ = 0.01.

Again, for Poisson arrival processes $D_{p}$ vanishes. Finally, we obtain an analytic expression for $D_{mp}$, which is given by

$$\begin{array}{c} D_{mp}=\frac{\beta (\mu_{p}-\mu_{m})}{\alpha (\alpha +\beta +\mu_{m})(\alpha +\beta +\mu_{p})}, \end{array}$$ (28)

and as expected, we note that $D_{mp}$ vanishes for Poisson arrivals and is negative for *μ*_*p*_ < *μ*_*m*_. In Fig 6, we have plotted the three analytic expressions together with simulation results as a function of *β*.

### Signatures for non-geometric bursts

As discussed in the previous section, it is widely assumed that the mRNA burst distribution can be represented by a conditional geometric distribution (i.e. including both single mRNA arrivals and geometrically distributed burst arrivals). While this assumption is consistent with multiple experimental observations, for general kinetic schemes the possibility of non-geometric mRNA burst distributions has to be considered. Thus, it is of interest to examine if the results obtained can be used to determine if the mRNA burst distribution deviates from a conditional geometric distribution.

To address the possibility of non-geometric mRNA burst distributions, let us first consider that the random variable corresponding to the mRNA burst distribution (*m*_*b*_) has a conditional geometric distribution. That is, the probability that a burst produces *n* mRNA molecules is given by

$$P\left(m_{b}=n\right)={\left(1-p\right)}^{n-1}p,$$ (29)

where 0 < *p* ≤ 1, and *n* = 1, 2, 3…∞. This distribution leads to

$$\sigma_{mb}^{2}=\left\langle m_{b}\right\rangle \left(\left\langle m_{b}\right\rangle -1\right).$$ (30)

Using Eqs (2) and (30) in Eq (4), and denoting $F_{m}=\sigma_{m_{s}}^{2}/\langle m_{s}\rangle$ as the Fano factor of mRNA copy number, Eq (4) can be rewritten as

$$F_{m}\left(\mu_{m}\right)=\frac{\left\langle m_{b}\right\rangle }{2}[1+K_{g}\left(\mu_{m}\right)].$$ (31)

Similarly, using the burst size distribution from Eq (29), the skewness in Eq (7) is given by

$$\begin{array}{ccc} \frac{\gamma_{m_{s}}\sigma_{m_{s}}^{3}}{\left\langle m_{s}\right\rangle } & = & 1+\left\langle m_{s}\right\rangle \left\langle m_{b}\right\rangle K_{1}\left(\mu_{m}\right)+2{\left\langle m_{b}\right\rangle }^{2}K_{2}\left(\mu_{m},\left\langle m_{b}\right\rangle \right)\\ & + & 2(\langle m_{b}\rangle -1)[(1+K_{3}\left(\mu_{m},\left\langle m_{b}\right\rangle \right))\left\langle m_{b}\right\rangle -1]. \end{array}$$ (32)

We note that Eq (32) connects experimentally measurable moments of the steady-state distribution to the parameters *K*_*g*_(*μ*_*m*_), *K*_*g*_(2*μ*_*m*_) and ⟨*m*_*b*_⟩. Furthermore, note that Eq (31) can be recast as *K*_*g*_(*μ*_*m*_) = (2*F*_*m*_(*μ*_*m*_)/⟨*m*_*b*_⟩)−1. Now, considering a change in the degradation rate from *μ*_*m*_ to 2*μ*_*m*_ (keeping the mean burst size, ⟨*m*_*b*_⟩ invariant), we obtain

$$K_{g}\left(2\mu_{m}\right)=\left(2F_{m}\left(2\mu_{m}\right)/\left\langle m_{b}\right\rangle \right)-1.$$ (33)

Using the above in Eq (32), we get an expression connecting experimentally measurable quantities associated with moments of the mRNA steady-state distribution. The resulting expression is:

$$\begin{array}{ccc} G_{m} & \equiv & \frac{\gamma_{m_{s}}\sigma_{m_{s}}^{3}{\left\langle m_{s}\right\rangle }^{-1}}{2F_{m}\left(2\mu_{m}\right)\left(\left\langle m_{s}\right\rangle -1\right)+F_{m}(1-2\left\langle m_{s}\right\rangle +2F_{m}\left(2\mu_{m}\right))}\\ & = & 1. \end{array}$$ (34)

We note that the above expression has been derived by making just one assumption, namely, the mRNA burst distribution is a conditional geometric distribution. The derived expression thus indicates that a combination of experimentally measurable quantities has to deviate from 1 if the mRNA burst distribution deviates from a conditional geometric distribution. Thus the analytical results derived provide a signature for deviation from conditional geometric mRNA bursts using measurements of the first three moments of the mRNA steady-state distribution.

The main requirement for using the above relation is that measurements of mRNA steady-state distribution can be carried out at two different rates of the mRNAs *μ*_*m*_ and 2*μ*_*m*_. Given that mRNA degradation rates can be tuned experimentally, a straightforward strategy to ensure that the degradation rate is tuned to twice the original value (2*μ*_*m*_) is to compare the mean mRNA levels at *μ*_*m*_ and 2*μ*_*m*_. Given these measurements, a value of $G_{m}\neq 1$ implies that bursts are not distributed geometrically. The strength of this result lies in the fact that it holds for general arrival processes for mRNA bursts with arbitrary waiting-time distributions.

Let us consider a specific simple model to illustrate the condition derived above. First, let the arrival process for mRNA bursts be a Poisson process. For this, arrival time distributions of mRNA bursts in the time domain, *t*, and in the Laplace domain, *s*, are given by

$$f\left(t\right)=k_{b}e^{-k_{b}t},\;\;\;\;\;f_{L}\left(s\right)=k_{b}/\left(k_{b}+s\right),$$ (35)

where *k*_*b*_ is the rate of arrival of mRNA bursts. For the mRNA burst distribution, let us assume that it is given by the negative binomial distribution, i.e.

$$P\left(m_{b}=n\right)=\frac{(n+r-1)!}{n!(r-1)!}p^{n}{\left(1-p\right)}^{r},$$ (36)

where 0 < *p* ≤ 1, *r* ≥ 1, and *n* = 0, 1, 2, 3…∞. For *r* = 1, the above reduces to the geometric distribution and therefore we expect *G*_*m*_ = 1 in this limit. Using the expressions for the moments derived in the previous section, we obtain an explicit expression for *G*_*m*_ (Supplementary S3 Text):

$$G_{m}=\frac{1}{3}(-\frac{p+1}{pr+1}+\frac{4}{p(r-1)+2}+2).$$ (37)

Notice that for the geometric bursts (*r* = 1) we get $G_{m}=1$, as expected. However, for non-geometric bursts, deviations of $G_{m}$ from 1 are observed (also see Fig S3–1 in Supplementary S3 Text). Two additional examples of microscopic models for non-geometric bursts (the two state random telegraph model and a model with three promoter states where mRNAs are produced from two states) are discussed in the.

The preceding analysis can be extended to protein steady-state distributions to derive a similar condition for deviations from geometric burst distributions in terms of steady state moments associated with proteins (see Supplementary S4 Text).

### Discussion

In this paper we study stochastic gene expression models with a general renewal-type arrival process for mRNAs. By mapping such a generic model of gene expression to systems studied in queueing theory, we derive analytical expressions for the moments for mRNA and protein steady-state distributions. While the focus of this work is on using approaches drawn from queueing theory, it is noteworthy that the kinetic scheme defined in Fig 1 can also be analyzed using the general theory of branching processes with immigration [58]. In future work, it would be of interest to explore potential connections between complementary approaches to such models based on branching processes and queueing theory.

While previous studies [37, 43] have focused on protein noise, in the present work we derive analytic expressions for higher order moments of both mRNA and protein steady-state distributions. For arbitrary kinetic schemes, the results obtained determine how the moments of steady-state distributions depend on model parameters. They elucidate how different sources (promoter-based regulation, transcriptional bursting, post-transcriptional regulation) combine to determine the overall noise and higher moments. Furthermore, the results derived show how parameters of interest (such as mean protein production rate *k*_*p*_) can be estimated for general models (i.e. without making any assumptions about specific features of the models).

The expressions derived for the moments can also be used to infer if the arrival process for mRNAs is non-Poisson or if the mRNA burst distribution deviates from the geometric distribution. Correspondingly, we obtain analytic conditions that provide signatures for non-Poisson arrivals of mRNA bursts and for non-geometric mRNA burst distributions. These conditions involve relations between combinations of of experimentally measurable quantities and can thus be tested by using measurements of either mRNA steady-state distributions or protein steady-state distributions or both. Apart from obtaining insights into the statistics of the arrival process, we can use the results derived for steady-state moments for accurately estimating burst parameters using an iterative approach. Notably, the results and the approaches developed in this work are valid for general models of gene expression i.e., given the general assumptions made, they do not depend on the specifics of the kinetic schemes.

It is important to note that the burst parameter estimation approaches presented in this paper rely on the accurate measurements of higher order moments, such as skewness or kurtosis. This, in turn requires that we have relatively large sample sizes. For example, simulations of two state random telegraph model (see Supplementary S5 Text) indicate that for the standard error in skewness to be below 10%, the sample size should be ∼ 1000. Current experimental limitations on measurements of mRNA distributions (e.g. using RNA FISH) do not allow for such large sample sizes and thus do not lead to accurate computation of skewness or kurtosis. While accurate measurements of higher moments are not readily available in the existing data, it is hoped that our results will provide motivation for carrying out the corresponding experiments in future. The combination of these experimental results with our theoretical approaches can be used in obtaining accurate representations of the arrival process and burst parameters for a wide range of cellular systems.

## Supporting Information

S1 Text Derivation of steady-state moments for mRNAs and proteins. (PDF)

S2 Text Condition for the two-state random telegraph model. (PDF)

S3 Text Illustrative examples for condition identifying non-geometric bursts. (PDF)

S4 Text Condition for non-geometric bursts using protein steady-state moments. (PDF)

S5 Text Estimation of error in skewness due to finite sample sizes. (PDF)
