# Supplementary material for: Transcriptional Bursting in Gene Expression: Analytical Results for General Stochastic Models
Source: PLoS Comput Biol. 2015 Oct 16;11(10):e1004292. doi: 10.1371/journal.pcbi.1004292 (PMC4608583; doi:10.1371/journal.pcbi.1004292)
Supplement: S1 Text — (PDF) [file pcbi.1004292.s001.pdf]

S1 Text: Derivation of steady-state moments for mRNAs and proteins

In this section, we discuss the derivation of expressions for the moments for mRNAs and protein steady-state distributions based on mapping of gene expression model to $GI^X/M/\infty$ model in the queuing theory. For $GI^X/M/\infty$ model, with λ and μ as the rates of customers mean arrival and service time respectively, exact steady state moments for the number of customers, N, can be obtained. Following [1], the binomial moments are given by

$$\left\langle \prod_{i=1}^{k} (X - i + 1) \right\rangle = G^k(1), \tag{S1-1}$$

where the symbol $\langle\ \rangle$ stands for average over many ensembles, $G^k(1)$ corresponds to k^{th} differentiation of $G(z)$ with respect to z at $z = 1$, with

$$G(z) = 1 + \sum_{r=1}^{\infty} B_r (z-1)^r.$$

The coefficients B_r are given as:

$$B_r = \lambda \sum_{k=1}^{r} \frac{A_k}{k!} B^{\star}_{r-k}(k\mu), \tag{S1-2}$$

where

$$B^{\star}_r(s) = \frac{f_L(s)}{1 - f_L(s)} \sum_{k=1}^{r} \frac{A_k}{k!} B^{\star}_{r-k}(s + k\mu), \tag{S1-3}$$

and $A_k = d^k A(z)/dz^k|_{z=1}$, with $A(z)$ as the generating function for the batch size distribution of arriving customers and $f_L(s)$ is the Laplace transform of arrival time distribution of customers, $f(t)$. Using $B^{\star}_0(s) = 1/s$, we can iteratively find all the B_r coefficients using Eqs. (S1-2),(S1-3) and hence all the moments from Eq. (S1-1). Using this procedure, expressions for the moments of number of customers can be obtained explicitly. For example, the corresponding mean, $\langle N\rangle$, variance, σ^2, and skewness γ, are given by:

$$\begin{aligned}
\langle N\rangle &= \frac{\lambda}{\mu} A_1, \\
\frac{\sigma^2}{\langle N\rangle^2} &= \frac{1}{\langle N\rangle}\left[1 + A_1\left(\frac{K_g(\mu) - 1}{2} + \frac{\lambda}{\mu}\right) + \frac{A_2}{2A_1} - \langle N\rangle\right], \\
\frac{\gamma\sigma^3}{\langle N\rangle} &= 1 + 2A_1^2\left(\frac{\lambda}{2\mu}\mathcal{K}_1(\mu) + \mathcal{K}_2(\mu, A_1)\right) + A_2\mathcal{K}_3(\mu, A_1) \\
&\quad + \frac{A_3}{3A_1},
\end{aligned} \tag{S1-4}$$

where $\mathcal{K}_1, \mathcal{K}_2$, and $\mathcal{K}_3$ are three functions given by

$$\begin{aligned}
\mathcal{K}_1(x) &= K_g(2x) - K_g(x), \\
\mathcal{K}_2(x, y) &= \frac{K_g(x) - 1}{4}\left(\frac{3}{y} + K_g(2x) - 1\right), \\
\mathcal{K}_3(x, y) &= \frac{3}{2y} + \frac{K_g(x) + K_g(2x)}{2} - 1,
\end{aligned} \tag{S1-5}$$

and $K_g(\mu)$ is the gestation factor,

$$K_g(\mu) = 1 + 2\left[\frac{f_L(\mu)}{1-f_L(\mu)} - \frac{\lambda}{\mu}\right], \quad \text{(S1-6)}$$

that encodes information about the arrival process. Extending this approach, we obtain expressions for higher moments. For example, fourth central moments are given by:

$$\begin{aligned}\langle (N-\langle N\rangle)^4\rangle &= 24B_4 + 36B_3 + 14B_2 + B_1 + 6B_1^2(B_1 \\ &+2B_2) - 4B_1\left(B_1 + 6(B_2+B_3)\right) - 3B_1^4,\end{aligned} \quad \text{(S1-7)}$$

where,

$$\begin{aligned}
B_1 &= \frac{\lambda}{\mu}A_1, \\
B_2 &= \frac{\lambda}{2\mu}\left(A_1^2\phi(\mu) + \frac{A_2}{2}\right), \\
B_3 &= \frac{\lambda}{3\mu}\left[\frac{A_3}{6} + \frac{A_1A_2}{2}\left(\phi(\mu)+\phi(2\mu)\right) + A_1^3\phi(\mu)\phi(2\mu)\right], \\
B_4 &= \frac{\lambda}{96\mu}\left[A_4 + 4A_1A_3\phi(3\mu) + 6A_2\phi(2\mu)\left(A_2 + 2A_1^2\phi(3\mu)\right)\right. \\
&+ \left. 4A_1\phi(\mu)\left\{A_3 + 3A_1A_2\phi(3\mu) + 3A_1\phi(2\mu)\left(A_2 + 2A_1^2\phi(3\mu)\right)\right\}\right]
\end{aligned} \quad \text{(S1-8)}$$

with $\phi(\mu)$ as

$$\phi(\mu) = \frac{f_L(\mu)}{1-f_L(\mu)}. \quad \text{(S1-9)}$$

Eqs.(S1-1),(S1-2) and (S1-3) can be used to derive steady state moments for mRNAs and proteins by mapping gene expression model to $GI^X/M/\infty$ model in queueing theory. To derive these expressions, we need to have the parameters associated with bursts statistics, A_k^m, A_k^p, $k = 1, 2, 3\ldots$, for both mRNAs and proteins, with superscripts 'm' and 'p' standing for mRNA and protein, respectively. For mRNAs, we note that the burst size parameters A_1, A_2 and A_3 are given by

$$\begin{aligned}
A_1^m &= \langle m_b\rangle, \\
A_2^m &= \langle m_b(m_b-1)\rangle, \\
A_3^m &= \langle m_b(m_b-1)(m_b-2)\rangle,
\end{aligned} \quad \text{(S1-10)}$$

where m_b is the mRNA burst size. Using Eq. (S1-10) in Eqs. (S1-4) and (S1-5), we can write explicit expressions for the first three moments of mRNAs copy numbers as written in the main text, which are exact for all parameter ranges.

To obtain corresponding burst size parameters for proteins (A_k^p), we note that each mRNA produces a random number of proteins, p_b. Using Eq. (1) in the main text, we can obtain expressions for the parameters A_k^p which are given by Eq. (10) in the main text. Corresponding expressions for the first three moments of protein copy numbers has been written in the main text. It is to be noted that resulting expressions for protein variance and skewness are exact in the burst limit (i.e. $\mu_m \gg \mu_p$), however, beyond this limit one can write approximate expressions for these quantities, as illustrated in the main text.

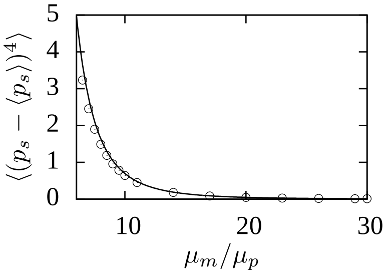

Figure S1-1. Steady state fourth central moment of proteins (scaled by 10^{-11}) for the model shown in Fig. 2a of the main text. Here lines represent analytic estimates while points correspond to the simulation results. Parameters are: $\alpha = 0.5$, $\beta = 0.25$, $k_m = 2$, $\langle m_b\rangle = 5$, $k_p = 0.5$.

It is also possible to obtain expressions for the fourth central moments of both mRNAs and proteins by using

$$A_4^m = \langle m_b(m_b - 1)(m_b - 2)(m_b - 3)\rangle \tag{S1-11}$$

for mRNAs and,

$$\begin{aligned} A_4^p \quad = \quad & 3\left[\langle m_b(m_b-1)\rangle\langle p_b\rangle\langle p_b(p_b-1)(p_b-2)\rangle + \langle m_b(m_b-1)\rangle \right. \\ & \left. \langle p_b(p_b-1)\rangle^2 + \langle m_b(m_b-1)(m_b-2)\rangle\langle p_b\rangle^2\langle p_b(p_b-1)\rangle\right] \\ & + \langle m_b(m_b-1)(m_b-2)(m_b-3)\rangle\langle p_b\rangle^4 \\ & + 3\langle m_b(m_b-1)(m_b-2)\rangle\langle p_b\rangle^2\langle p_b(p_b-1)\rangle \\ & + \langle m_b(m_b-1)\rangle\langle p_b\rangle\langle p_b(p_b-1)(p_b-2)\rangle \\ & + \langle m_b\rangle\langle p_b(p_b-1)(p_b-2)(p_b-3)\rangle, \end{aligned} \tag{S1-12}$$

for proteins, by using Eq. (1) in the main text. Again, the fourth moment for mRNAs is exact for the entire parameter regime, while for proteins the expression is exact only in the burst limit, i.e. $\mu_m \gg \mu_p$. Beyond the burst limit, we can find approximate expressions for the fourth moment using the approach outlined for the second and third moments, i.e. comparing our exact result in the burst-limit with the exact results obtained for the two-stage model [2]. This leads to the corresponding scaling of the coefficients A_k^p, specifically:

$$\begin{aligned} A_2^p &\to A_2^p \frac{1}{1 + \frac{\mu_p}{\mu_m}}, \\ A_3^p &\to A_3^p \frac{1}{(1 + \frac{\mu_p}{\mu_m})(1 + 2\frac{\mu_p}{\mu_m})}, \\ A_4^p &\to A_4^p \frac{1}{(1 + \frac{\mu_p}{\mu_m})(1 + 2\frac{\mu_p}{\mu_m})(1 + 3\frac{\mu_p}{\mu_m})}. \end{aligned} \tag{S1-13}$$

As shown in Fig.S1-1 the resulting analytical expression shows good agreement with results from simulations.

References

1. Liu L, Kashyap BRK, Templeton JGC. On the GIX/G/Infinity system. Jour Appl Prob. 1990;27(3):671–683.

2. Bokes P, King JR, Wood AT, Loose M. Exact and approximate distributions of protein and mRNA levels in the low-copy regime of gene expression. Journal of mathematical biology. 2012;64(5):829–854.
