# Supplementary material for: Transcriptional Bursting in Gene Expression: Analytical Results for General Stochastic Models
Source: PLoS Comput Biol. 2015 Oct 16;11(10):e1004292. doi: 10.1371/journal.pcbi.1004292 (PMC4608583; doi:10.1371/journal.pcbi.1004292)
Supplement: S2 Text — (PDF) [file pcbi.1004292.s002.pdf]

S2 Text: Condition for the two-state random telegraph model

In this section, we derive analytic conditions for validating the proposition that the underlying kinetic scheme can be represented by a two-state random telegraph model. For this model, the form $g_L(s) = g_1^0(s) = 1/(1 + b_1 s)$ in the main text with $g_L(s)$ as the phase-type process, given by Eq.(19) of the main text, is exact. Using the first three mRNA moments, we can estimate the parameters b_1, β, and k_m in terms these moments, and plugging these into the equation for the fourth moment, we derive:

$$\mathcal{C}_{rt} \equiv \frac{\nu_4^m \left(F_m(4F_m + 2\langle m_s\rangle - 5) - 2\langle m_s\rangle - \frac{\nu_3^m}{\langle m_s\rangle} + 2 \right)}{\langle m_s\rangle \left(\xi_0 + \xi_1 F_m + \xi_2 F_m^2 + \xi_3 F_m^3\right)} = 1. \qquad \text{(S2-1)}$$

where $\nu_3^m = \langle (m_s - \langle m_s\rangle)^3\rangle$ and $\nu_4^m = \langle (m_s - \langle m_s\rangle)^4\rangle$ are the third and fourth central moments associated with mRNA measurements respectively, and ξ_0, ξ_1, ξ_2 and ξ_3 are functions of $\langle m_s\rangle$ and ν_3^m:

$$\begin{aligned}
\xi_0 &= 3\left(\frac{\nu_3^m}{\langle m_s\rangle}\right)^2 (\langle m_s\rangle - 3) + 6\frac{\nu_3^m}{\langle m_s\rangle}(\langle m_s\rangle - 1),\\
\xi_1 &= 3\left(\frac{\nu_3^m}{\langle m_s\rangle}\right)^2 + (11 - 18\langle m_s\rangle)\frac{\nu_3^m}{\langle m_s\rangle} + 4(\langle m_s\rangle - 1),\\
\xi_2 &= 3\left(\frac{\nu_3^m}{\langle m_s\rangle}\right)(2 + \langle m_s\rangle) - (6\langle m_s\rangle + 13)\langle m_s\rangle + 16,\\
\xi_3 &= 6\langle m_s\rangle^2 + 15\langle m_s\rangle - 17. \qquad \text{(S2-2)}
\end{aligned}$$

Thus once we have measurements of the first four moments associated with mRNA, then Eq.(S2-1) must be satisfied if the underlying kinetic scheme is a two-state random telegraph model. That is, Eq.(S2-1) provides a prescription for the validity of two-state random telegraph model that can be tested experimentally.

For the sake of illustration, let us consider a kinetic scheme with $g_L(s) = (\alpha/(\alpha + s))^n$, which represents the presence of n identical kinetic steps, each with rate α, during the transition of gene from OFF to ON. Using Eqs. (2),(4),(7) and (23) we can explicitly find its moments that can be used in Eq. (S2-1) to get C_{rt}. As expected, we can see that, in Fig. S2-1, for $n = 1$, we get $C_{rt} = 1$ and deviation from this value is evident for $n > 1$.

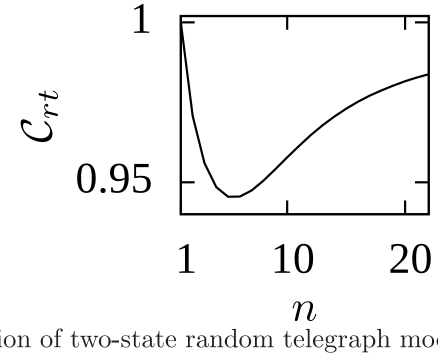

Figure S2-1. Verification of two-state random telegraph model as the underlying kinetic scheme: C_{rt} as a function of number of steps, n, for transitions from *off* to *on* state. Parameters: $\alpha = \beta = 2$, $k_m = 100$, $\mu = 1$.
