# Supplementary material for: Transcriptional Bursting in Gene Expression: Analytical Results for General Stochastic Models
Source: PLoS Comput Biol. 2015 Oct 16;11(10):e1004292. doi: 10.1371/journal.pcbi.1004292 (PMC4608583; doi:10.1371/journal.pcbi.1004292)
Supplement: S3 Text — (PDF) [file pcbi.1004292.s003.pdf]

S3 Text: Illustrative examples for condition identifying non-geometric bursts

In this section, we consider illustrative examples for the condition relating to the assumption of geometric burst distribution for mRNAs.

Poisson arrival of negative binomial bursts

For Poisson arrival of negative binomial bursts, given by Eqs. (35) and (36) in the main text, let us first consider the steady state expressions for the moments. Using Eq. (36) we note that,

$$\begin{aligned} \langle m_b \rangle &= \frac{pr}{1-p}, \\ \langle m_b(m_b-1) \rangle &= \frac{p^2 r(r+1)}{(p-1)^2}, \\ \langle m_b(m_b-1)(m_b-2) \rangle &= -\frac{p^3 r(r+1)(r+2)}{(p-1)^3}. \end{aligned} \tag{S3-1}$$

Plugging these values in Eqs. (2),(4) and (7) of main text and making use of Eq. (35) for $f_L(s)$, we obtain the expression for the steady state moments. For example, mean number of mRNAs can be written as

$$\langle m_s \rangle = \frac{k_b pr}{\mu_m (1-p)}, \tag{S3-2}$$

its Fano factor as

$$F_m = \frac{p(r-1)+2}{2(1-p)}, \tag{S3-3}$$

and its skewness as

$$\frac{\gamma_{m_s} \sigma^3_{m_s}}{\langle m_s \rangle} = \frac{p(p(r-1)(2r-1)+9r-3)+6}{6(p-1)^2}. \tag{S3-4}$$

Using these moments in Eq. (34), we get an explicit expression for $\mathcal{G}_m$:

$$\mathcal{G}_m = \frac{1}{3}\left(-\frac{p+1}{pr+1} + \frac{4}{p(r-1)+2} + 2\right), \tag{S3-5}$$

as written in the main text. We notice that for the geometric bursts ($r = 1$) we get $\mathcal{G}_m = 1$, as expected. However, away from this limit ($r = 1$), deviations of G_m values away from 1 can be seen, see Fig. S3-1.

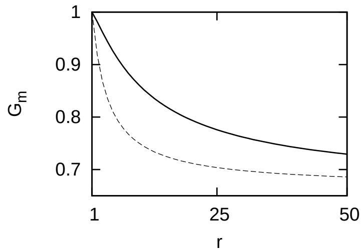

Figure S3-1. $\mathcal{G}_m$ as a function of r for two different values of p, 0.25 (solid line) and 0.75 (dashed line).

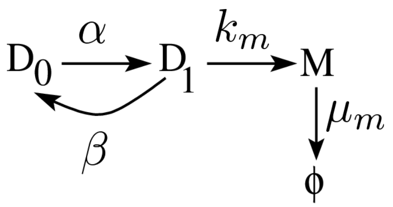

Figure S3-2. Schematic representation for the transcriptional kinetic scheme of two state model. Gene in OFF state (D_0) switches to ON state (D_1) with rate α and can switch back to OFF state with rate β. When the gene is ON, it produces mRNA bursts with rate k_m, and mRNAs can then degrade with rate μ_m.

Two-state random telegraph model

Next, we consider the two-state random telegraph model, a widely used model for gene expression, Fig. S3-2. Here the gene switches stochastically between its ON and OFF states: the rate of switching from ON to OFF is α while that from OFF to ON it is β. Gene in the ON state then produces a single mRNA with rate k_m, which can degrade further with rate μ_m. To verify our condition for geometric bursts, the first step is to find mRNA moments, mean, Fano factor and skewness. However, as can be seen in Eqs. (4) and (7), to find these moments the central quantity that needs to be evaluated is $f_L(s)$, the waiting time distribution for the arrival of mRNA bursts in the Laplace domain. Equivalently, this waiting time distribution translates into finding the first passage time distribution for the production of mRNA given that gene is in the active state D_1 at time $t = 0$. If $P_0(t)$ and $P_1(t)$ denote the probabilities of gene being in OFF and ON states at time t, respectively, then the first passage time distribution is given by,

$$f(t) = k_m P_1(t), \tag{S3-6}$$

where the probabilities, $P_0(t)$ and $P_1(t)$ obey the Master equation

$$\begin{aligned} \frac{dP_0(t)}{dt} &= \beta P_1(t) - \alpha P_0(t), \\ \frac{dP_1(t)}{dt} &= \alpha P_0(t) - \beta P_1(t). \end{aligned} \tag{S3-7}$$

The corresponding evolution equation in the Laplace domain is given by

$$\begin{aligned} sf_0(s) - x_0 &= \beta f_1(s) - \alpha f_0(s), \\ sf_1(s) - y_0 &= \alpha f_0(s) - \beta f_1(s), \end{aligned} \tag{S3-8}$$

where $f_j(s)$ stands for the Laplace transform of $P_j(t)$, and x_0 and y_0 are the initial values of P_0 and P_1, respectively. For the process in the Fig. S3-2, where mRNAs are always produced from the active state, we take $P_0 = 0$ and $P_1 = 1$, and obtain the Laplace transform of first passage waiting time distributions as

$$f_L(s) = \frac{k_m(\alpha + s)}{s^2 + s(\alpha + \beta + k_m) + \alpha k_m}. \tag{S3-9}$$

Using this $f_L(s)$ in Eqs. (2),(4) and (7), we obtain explicit expressions for the first three moments of mRNA copy numbers:

$$\begin{aligned}
\langle m_s \rangle &= \left(\frac{\alpha}{\alpha+\beta}\right)\frac{k_m}{\mu_m}, \\
F_m &= 1 + \frac{\beta k_m}{(\alpha+\beta)(\mu_m+\alpha+\beta)}, \\
\frac{\gamma_{m_s}\sigma^3_{m_s}}{\langle m_s \rangle} &= \frac{1}{(\alpha+\beta)^2(\mu_m+\alpha+\beta)(2\mu_m+\alpha+\beta)}\big[\alpha^4+ \\
& 4\alpha^3\beta+\beta^2(k_m+\beta)(2k_m+\beta)+2\mu_m^2(\alpha+\beta)^2 \\
& +3\alpha^2\beta(k_m+2\beta)+2\alpha\beta(-{k_m}^2+3k_m\beta+2\beta^2) \\
& +3\mu_m(\alpha+\beta)(2k_m\beta+(\alpha+\beta)^2)\big]
\end{aligned} \tag{S3-10}$$

Using these values of mean, Fano factor and skewness in Eq. (34), we get $\mathcal{G}_m = 1$, as expected.

Transcription from two promoter states

Finally, we consider a model as shown in Fig. S3-3. Here D_0, D_1 and D_2 are three promoter states. Now, instead of having mRNA production from just a single state, as discussed above, let us assume that mRNAs are produced by two states D_1 and D_2 with rates k_{m1} and k_{m2}, respectively. In the absence of any one of these two transcriptional routes, bursts are geometrically produced as discussed above. However, when both transcriptional routes are present we expect deviation from $\mathcal{G}_m = 1$, which we show in the following.

To start with, let us first denote by $P_\sigma(m,t)$ as the probability that there are m number of mRNAs at a time t in the promoter state $\sigma = 0, 1, 2$. The evolution of these probabilities reads as

$$\begin{aligned}
\frac{P_0(m,t)}{dt} &= \mu_m(m+1)P_0(m+1,t)+\beta_1 P_2(m,t) \\
&- (\alpha+\mu_m m)\,P_0(m,t), \\
\frac{P_1(m,t)}{dt} &= \alpha P_0(m,t)+k_{m1}P_1(m-1,t)+\mu_m(m+1) \\
& P_1(m+1,t)-(\beta_2+k_{m1}+\mu_m m)P_1(m,t), \\
\frac{P_2(m,t)}{dt} &= \beta_2 P_1(m,t)+k_{m2}P_2(m-1,t)+\mu_m(m+1) \\
& P_2(m+1,t)-(\beta_1+k_{m2}+\mu_m m)\,P_2(m,t).
\end{aligned} \tag{S3-11}$$

In the following, we will use this equation to get the first three moments of mRNA in the steady state. Let us first sum over all possible values of m and use the normalization $\sum_\sigma P_\sigma(m) = 1$. This leads to

$$\begin{aligned}
P_0 &= \frac{\beta_1\beta_2}{\beta_1\beta_2+\alpha(\beta_1+\beta_2)}, \\
P_1 &= \frac{\alpha\beta_1}{\beta_1\beta_2+\alpha(\beta_1+\beta_2)}, \\
P_2 &= \frac{\alpha\beta_2}{\beta_1\beta_2+\alpha(\beta_1+\beta_2)}.
\end{aligned} \tag{S3-12}$$

Next, multiplying Eq. (S3-11) by m and summing over all m, we have

$$\begin{aligned}
&\beta_1\langle m\rangle_2 - (\mu_m + \alpha)\langle m\rangle_0 = 0,\\
&\alpha\langle m\rangle_0 + k_{m1}P_1 - (\mu_m + \beta_2)\langle m\rangle_1 = 0,\\
&\beta_2\langle m\rangle_1 + k_{m2}P_2 - (\mu_m + \beta_1)\langle m\rangle_2 = 0,
\end{aligned} \tag{S3-13}$$

where $\langle m\rangle_\sigma = \sum_m mP_\sigma(m)$. These equations are solved to get the mean number of mRNAs as

$$\langle m\rangle = \sum_\sigma \langle m\rangle_\sigma = \frac{k_{m1}P_1 + k_{m2}P_2}{\mu_m}. \tag{S3-14}$$

Similarly, if we multiply Eq. (S3-11) by m^2 and sum over all m, and denote $\langle m^2\rangle_\sigma = \sum_m m^2 P_\sigma(m)$, we get

$$\begin{aligned}
&\beta_1\langle m^2\rangle_2 + \mu_m\langle m\rangle_0 - (2\mu_m + \alpha)\langle m^2\rangle_0 = 0,\\
&\alpha\langle m^2\rangle_0 - (\beta_2 + 2\mu_m)\langle m^2\rangle_1 + (2k_{m1} + \mu_m)\langle m\rangle_1\\
&+k_{m1}P_1 = 0,\\
&\beta_2\langle m^2\rangle_1 - (\beta_1 + 2\mu_m)\langle m^2\rangle_2 + (\mu_m + 2k_{m2})\langle m\rangle_2\\
&+k_{m2}P_2 = 0,
\end{aligned} \tag{S3-15}$$

which can be solved to get $\langle m^2\rangle = \sum_\sigma \langle m^2\rangle_\sigma$. Finally, to get third moment we multiply Eq. (S3-11) by m^3 and sum over m, the resulting equations read

$$\begin{aligned}
&\mu_m\left[-3\langle m^3\rangle_0 + 3\langle m^2\rangle_0 - \langle m\rangle_0\right] + \beta_1\langle m^3\rangle_2 - \alpha\langle m^3\rangle_0 = 0,\\
&\alpha\langle m^3\rangle_0 + k_{m1}\left[3\langle m^2\rangle_1 + 3\langle m\rangle_1 + P_1\right]\\
&+\mu_m\left[-3\langle m^3\rangle_1 + 3\langle m^2\rangle_1 - \langle m\rangle_1\right] - \beta_2\langle m^3\rangle_1 = 0,\\
&\beta_2\langle m^3\rangle_1 + k_{m2}\left[3\langle m^2\rangle_2 + 3\langle m\rangle_2 + P_2\right]\\
&+\mu_m\left[-3\langle m^3\rangle_2 + 3\langle m^2\rangle_2 - \langle m\rangle_2\right] - \beta_1\langle m^3\rangle_2 = 0,
\end{aligned}$$

which are solved to get the third moment of mRNAs. Once we have the first three moments, we can evaluate $\mathcal{G}_m$ using Eq. (34). The resulting expression is somewhat complicated, and therefore we just show the result in Fig. S3-3. As can be seen, for a given set of other parameters, variations of $\mathcal{G}_m$ with k_{m2} show that it approaches 1 for $k_{m2} = 0$, as expected. However, beyond this significant deviations are visible.

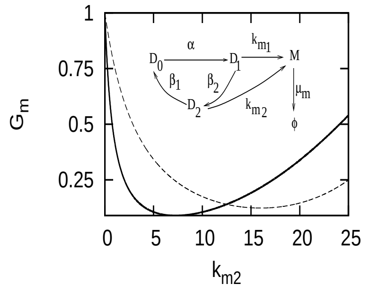

Figure S3-3. Variation of $\mathcal{G}_m$ as a function of transcriptional rate k_{m2} has been shown for two different values of α, 1(solid line) and 2(dashed line), for the model (inset). Other parameters are: $\beta_1 = 0.5, \beta_2 = 0.25, k_{m1} = 40, \mu_m = 1$.
