# Supplementary material for: Transcriptional Bursting in Gene Expression: Analytical Results for General Stochastic Models
Source: PLoS Comput Biol. 2015 Oct 16;11(10):e1004292. doi: 10.1371/journal.pcbi.1004292 (PMC4608583; doi:10.1371/journal.pcbi.1004292)
Supplement: S4 Text — (PDF) [file pcbi.1004292.s004.pdf]

S4 Text: Condition for non-geometric bursts using protein steady-state moments

The condition for geometric bursts derived in the paper is an exact condition using mRNA steady state measurements, however, it is of interest to see if a similar condition can be obtained using moments of the protein steady-state distribution. As we show below, this indeed can be done in the limit that the mRNA degradation rate is very large compared to that of protein (which is a valid approximation in many cellular systems). In this so called burst limit, we can derive analytical expressions for the first three moments of the protein steady-state distribution in the burst limit as discussed in the main text (Eqs. (2), (6), and (9)). In the burst limit, for geometrically distributed mRNA and protein bursts, the variance in protein copy numbers, $\sigma^2_{p_s} = \langle p_s^2 \rangle - \langle p_s \rangle^2$, is written as

$$\frac{\sigma^2_{p_s}}{\langle p_s \rangle^2} = \frac{1}{\langle p_s \rangle} \left[1 + \frac{b}{2} \left(1 + K_g(\mu_p) \right) \right], \tag{S4-1}$$

and similarly, skewness is given by

$$\frac{\gamma_{p_s} \sigma^3_{p_s}}{\langle p_s \rangle} = 1 + 2b^2 \left[\frac{\langle p_s \rangle}{2b} \mathcal{K}_1(\mu_p) + \mathcal{K}_2(\mu_p, b) + \mathcal{K}_3(\mu_p, b) + 1 \right]. \tag{S4-2}$$

Using Eq. (S4-1) in (S4-2) leads to

$$\begin{aligned} \mathcal{G}_p &\equiv \frac{\gamma_{p_s} \sigma^3_{p_s} \langle p_s \rangle^{-1}}{1 + 2\langle p_s \rangle \left(F_p(2\mu_p) - F_p \right) + (F_p - 1)(1 + 2F_p(2\mu_p))} \\ &= 1 \end{aligned} \tag{S4-3}$$

This is the condition for the protein burst distribution to be geometric. Note that if the protein burst distribution is geometric, this implies that the underlying mRNA burst distribution is a conditional geometric distribution [1]. Thus the value of $\mathcal{G}_p \neq 1$ indicates that both protein and mRNA burst distributions differ from the geometric distribution.

Next, we verify this condition for the two-state random telegraphic model of Fig. 2a, with each transcription event leading to the arrival of just one mRNA instead of arrival of conditionally geometric bursts. For this, we first note that, using Eqs. (2),(6), (9) and (S3-9), protein moments are given by

$$\begin{aligned} \langle p_s \rangle &= \left(\frac{\alpha}{\alpha + \beta} \right) \frac{k_m k_p}{\mu_m \mu_p}, \\ F_p &= 1 + \frac{k_p}{\mu_m} \left[1 + \frac{k_m \beta}{(\alpha + \beta)(\mu_p + \alpha + \beta)} \right], \\ \frac{\gamma_{p_s} \sigma^3_{p_s}}{\langle p_s \rangle} &= \frac{1}{\xi(\mu_p + \xi)(\mu_p - \xi)^2 \mu_m^2} [(k_p + \mu_m)(2k_p + \mu_m) \\ &\quad \xi(\mu_p + \xi)(\mu_p - \xi)^2 - k_m k_p (\mu_p - \xi)(3\mu_m(\mu_p + \xi) \\ &\quad + 2k_p(\mu_p + 2\xi) - 2k_m k_p)\beta + 4k_m^2 k_p^2 \beta^2] \end{aligned} \tag{S4-4}$$

where

$$\xi = \alpha + \beta + \mu_p.$$

Using this in Eq. (S4-3), we get $\mathcal{G}_p = 1$ which is consistent with the fact that the protein bursts distribution is geometric.

References

1. Elgart V, Jia T, Fenley AT, Kulkarni R. Connecting protein and mRNA burst distributions for stochastic models of gene expression. Physical biology. 2011;8(4):046001.
