# Supplementary material for: Transcriptional Bursting in Gene Expression: Analytical Results for General Stochastic Models
Source: PLoS Comput Biol. 2015 Oct 16;11(10):e1004292. doi: 10.1371/journal.pcbi.1004292 (PMC4608583; doi:10.1371/journal.pcbi.1004292)
Supplement: S5 Text — (PDF) [file pcbi.1004292.s005.pdf]

S5 Text: Estimation of error in skewness due to finite sample sizes

To determine how the error in skewness varies with finite sample size (n), we performed simulations for the random telegraph model as shown in Fig.2a. For a fixed sample size, we generated a probability distribution corresponding to the steady-state values of mRNA skewness and obtained the corresponding standard error, $\sigma_{\gamma_{m_s}}$. Repeating this for different sample sizes, we obtain the variations in the standard error of skewness with the number of samples. In Fig.S5-1, we show the corresponding results. As expected, $\sigma_{\gamma_{m_s}}/\gamma_{m_s}$ decreases monotonically with increasing values of sample size.

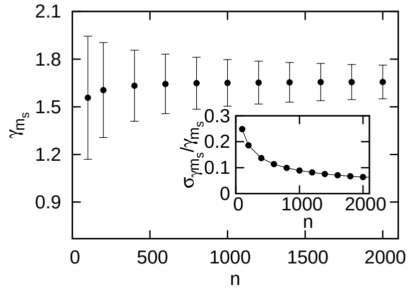

Figure S5-1. Variations in skewness of the mRNA steady-state distribution for the model in Fig.2a as a function of sample size n, with bars representing the standard error. Inset: Variations of $\sigma_{\gamma_{m_s}}/\gamma_{m_s}$ with the sample size with parameters as: $\alpha = 1$, $\beta = 2$, $k_m = 2$, $\mu_m = 1$.
